# From Local to Systemic: The Journey of Tick Bite Biomarkers in Australian Patients

**DOI:** 10.3390/ijms26041520

**Published:** 2025-02-11

**Authors:** Wenna Lee, Amanda D. Barbosa, Amy Huey-Yi Lee, Andrew Currie, David Martino, John Stenos, Michelle Long, Miles Beaman, Nathan T. Harvey, Nina Kresoje, Patrycja Skut, Peter J. Irwin, Prasad Kumarasinghe, Roy A. Hall, Rym Ben-Othman, Stephen Graves, Tobias R. Kollmann, Charlotte L. Oskam

**Affiliations:** 1 Centre for Biosecurity and One Health, Harry Butler Institute, Murdoch University, Murdoch, WA 6150, Australia; 2 The Kids Research Institute Australia, Nedlands, WA 6009, Australiatkollm@mac.com (T.R.K.); 3 School of Medical, Molecular, and Forensic Sciences, College of Environmental and Life Sciences, Murdoch University, Murdoch, WA 6150, Australia; 4 UWA Medical School, University of Western Australia, Crawley, WA 6009, Australia; 5 School of Veterinary Medicine, College of Environmental and Life Sciences, Murdoch University, Murdoch, WA 6150, Australia; 6 CAPES Foundation, Ministry of Education of Brazil, Brasilia-DF 70040-020, Brazil; 7 Department of Molecular Biology and Biochemistry, Simon Fraser University, Burnaby, BC V5A 1S6, Canada; 8 Personalised Medicine Centre, Health Futures Institute, Murdoch University, Murdoch, WA 6150, Australia; 9 Wal-yan Respiratory Research Centre, The Kids Research Institute Australia, Nedlands, WA 6009, Australia; 10 Australian Rickettsial Reference Laboratory, Barwon Biomedical Research, University Hospital Geelong, Barwon Health, Geelong, VIC 3220, Australia; 11 Faculty of Health and Medical Sciences, Pathology & Laboratory Medicine, University of Western Australia, Crawley, WA 6009, Australia; 12 PathWest Laboratory Medicine, Department of Anatomical Pathology, QEII Medical Centre, Nedlands, WA 6009, Australia; 13 School of Medicine, University of Western Australia, Crawley, WA 6009, Australia; 14 College of Science, Health, Education and Engineering, Murdoch University, Murdoch, WA 6150, Australia; 15 Western Dermatology, Hollywood Medical Centre, Nedlands, WA 6009, Australia; 16 Australian Infectious Diseases Research Centre, School of Chemistry and Molecular Biosciences, The University of Queensland, St Lucia, QLD 4072, Australia; 17 RAN BioLinks Ltd., 10212 Yonge Street, 202, Richmond Hill, ON L4C 3B6, Canada; 18 Department of Microbiology & Immunology, Faculty of Medicine, Dalhousie University, Halifax, NS B3H 4R2, Canada

**Keywords:** emerging diseases, MULTI-OMICS, systems biology, tick-borne diseases

## Abstract

Tick bites and tick-related diseases are on the rise. Diagnostic tests that identify well-characterised tick-borne pathogens (TBPs) possess limited capacity to address the causation of symptoms associated with poorly characterised tick-related illnesses, such as debilitating symptom complexes attributed to ticks (DSCATT) in Australia. Identification of local signals in tick-bitten skin that can be detected systemically in blood would have both clinical (diagnostic or prognostic) and research (mechanistic insight) utility, as a blood sample is more readily obtainable than tissue biopsies. We hypothesised that blood samples may reveal signals which reflect relevant local (tissue) events and that the time course of these signals may align with local pathophysiology. As a first step towards testing this hypothesis, we compared molecular signatures in skin biopsies taken from the tick-bite location of human participants, as published in our previous study, together with peripheral blood signatures obtained concurrently. This approach captures differentially expressed molecules across multiple omics datasets derived from peripheral blood (including cellular and cell-free transcriptomics, proteomics, metabolomics, and DNA methylation), and skin biopsies (spatial transcriptomics). Our original data revealed that extracellular matrix organisation and platelet degranulation pathways were upregulated in the skin within 72 h of a tick bite. The same signals appeared in blood, where they then remained elevated for three months, displaying longitudinally consistent alterations of biological functions. Despite the limited sample size, these data represent proof-of-concept that molecular events in the skin following a tick bite can be detectable systemically. This underscores the potential value of blood samples, akin to a liquid biopsy, to capture biomarkers reflecting local tissue processes.

## 1. Introduction

The escalating prevalence and public health threat posed by tick bites calls for innovative approaches that would enhance our understanding of disease processes [1]. The need for suitable biomarkers is underscored by the difficulty of diagnosing tick-borne diseases (TBDs) since patients may exhibit a wide range of symptoms yet show ’normal’ test results using current diagnostic methods [2]. Starting from the assumption that TBDs ultimately arise from the tick bite site, we hypothesised that samples such as blood may allow the detection of biological signals that reflect the pathophysiological events originating from the tissue at that site. Such liquid biopsy methods have revolutionised disease detection and monitoring in prenatal testing and cancer diagnostics [3,4,5,6]. Importantly, interrogating and analysing molecular biomarkers present in a bodily fluid such as blood have allowed for the identification of biomarkers as promising monitoring tools, offering minimally invasive access to diseased tissue and near real-time insights into disease status and progression [3,4,5,6]. We reasoned that applying liquid biopsy methodologies could potentially lead not only to improved understanding and earlier diagnosis of TBDs but also to improved treatment outcomes [1,5].

We designed a multi-omics study to identify both single omics that capture the most expressed differences as well as an integrative method that enables different omics to complement each other to enhance the robustness of our findings. To assess the feasibility of a liquid biopsy approach to capture disease progression for TBDs, tick-bitten skin biopsies (solid biopsies) and their contralateral controls were collected concurrently with peripheral blood samples (liquid biopsies) at time points of; (i) enrolment, (ii) one week, and (iii) three months after tick bite. Multi-omics analysis of blood samples was performed at all three time points, encompassing cellular and cell-free transcriptomics, proteomics, metabolomics, and epigenetics (DNA methylation) to ascertain the most informative omics capable of capturing disease progression. The multi-omics data generated in this study were compared with the spatial transcriptomics data we reported previously (Lee. et al., 2024) [7]. Notably, two of these—cell-free transcriptomics and proteomics—hold particular potential as liquid biopsy modalities as they are known to be capable of detecting perturbations from within cancerous tissue [8]. Specifically, four genes—LRIG3, WIF1, LIN7B and RRM2—associated with processes such as cell growth regulation, signalling pathways, and immune responses [9] were identified as differentially expressed in both skin and blood samples.

## 2. Results

Six participants were recruited in Perth, Western Australia, in accordance with the inclusion criteria previously described [7]. Participant details (Table S1) (post quality assurance and control, processing, and normalisation) (Figure S1) are described in Lee et al., 2024. All participants had been bitten by ticks identified as *Amblyomma triguttatum* (the ornate kangaroo tick), with instars recorded (1 × larva, 3 × nymphs, and 2 × adults (1 × male and 1 × female)). This native Australian tick is well known to feed on humans opportunistically [10].

### 2.1. Local (Solid Biopsy) Signatures with Spatial Transcriptomics

We have previously published the analysis of the spatial (tissue) transcriptomic data [7]. We here include these data but amended to support integration with the systemic blood-based (liquid biopsy) data sets.

**Figure 1:**
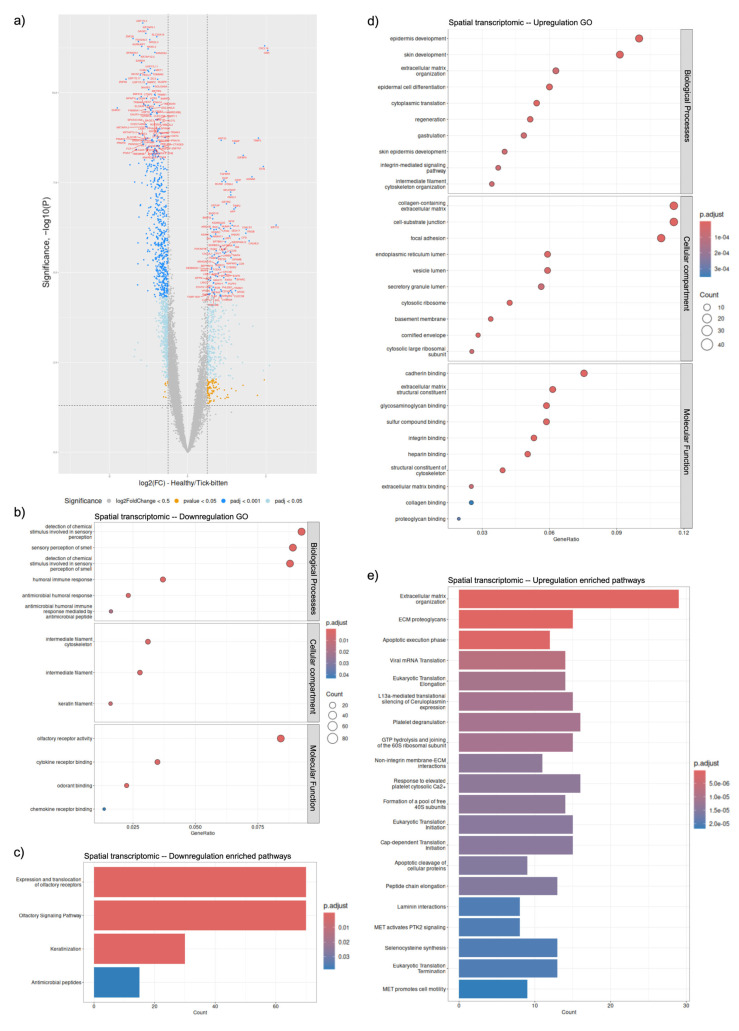
Differential expressed gene analysis comparing transcripts of tick-bitten skin to contralateral controls. (**a**) Volcano plot of differentially expressed genes (DEGs), identified with *p*-value < 0.05 and fold change of 1.5 in either direction. Grey: Not significant or under a log2(FC) < 0.5; Yellow: *p*-value < 0.05; Cyan: False discovery rate adjusted *p*-value < 0.05; Blue: False discovery rate adjusted *p*-value < 0.001; (**b**) Dotplot of downregulated spatial DEGs in terms of significance in cellular compartment, molecular function, and biological processes. The size of the dots represents the number of genes within the enriched pathways while the dot colour represents the enrichment scores based on adjusted *p*-values; (**c**) Enriched pathway analysis of downregulated spatial DEGs in terms of significance based on adjusted *p*-value represented with the bar colour; (**d**) Dotplot of upregulated spatial DEGs in terms of significance in the cellular compartment, molecular function, and biological processes. The size of the dots represents the number of genes within the enriched pathways while the dot colour represents the enrichment scores based on adjusted *p*-values; (**e**) Enriched pathway analysis of upregulated spatial DEGs in terms of significance based on adjusted *p*-value, represented with the bar colour.

There were 1380 differentially expressed genes (DEGs) identified (Figure 1a); 1018 were downregulated and 362 were upregulated when the transcripts in all tick-bitten skin sections were compared to those obtained from contralateral control skin sections. All DEGs and adjusted *p*-values are listed in Table S1. The most significant downregulated spatial DEGs were ELOVL5 (which translates to a membrane protein localised to the endoplasmic reticulum associated with elongation of the long-chain polyunsaturated fatty acids) with an adjusted *p*-value of 4.82 × 10^−10^ and TOMMs (a component of the mitochondrial outer membrane translocase complex) with an adjusted *p*-value of 2.02 × 10^−9^. Among the downregulated spatial DEGs, olfactory receptor activity (GO ID:0004984) was the most prominent molecular function, with the detection of chemical stimulus involved in sensory perception of smell (GO ID:0050911) as the most significant of the biological processes and intermediate filament cytoskeleton (GO ID:0045111) as the most significant cellular compartment these DEGs were localised to (Figure 1b). All GO results and the adjusted *p*-values are listed in Table S2. The pathway analysis with the identified downregulated spatial DEGs identified four significant pathways. “Expression and translocation of olfactory receptors” and “olfactory signalling pathway”, both comprising 70 DEGs, were identified as the top two most significant pathways derived from the downregulated DEGs, with adjusted *p*-values of 1.03 × 10^−17^ and 1.75 × 10^−17^, respectively (Figure 1c). All pathways identified and the adjusted *p*-values are listed in Table S3.

For spatially upregulated DEGs, the most significant molecular function was associated with extracellular matrix structural constituent (GO ID:0005201) with an adjusted *p*-value of 6.96 × 10^−10^, while cadherin binding (GO ID:0045296) with an adjusted *p*-value of 7.80 × 10^−8^ had the highest gene count of 27 (Figure 1d). The most prominent biological process implicated was epidermis development (GO ID:0008544), and the collagen-containing extracellular matrix (GO ID:0062023) was the most significant cellular compartment where these DEGs were localised (Figure 1d). The most significantly upregulated spatial DEGs were CXCL14 (a cytokine gene involved in inflammatory and immune processes) and GSN (coding for an actin-binding protein with multiple isoforms) with adjusted *p*-values of 5.69 × 10^−9^ and 6.46 × 10^−9^, respectively. An overexpression analysis (ORA) was carried out with the upregulated spatial DEGs and identified 67 significant pathways, listed in Table S3. The most significant pathways were “extracellular matrix organisation” comprising 29 DEGs and “ECM proteoglycans” comprising 15 DEGs, both with adjusted *p*-values of 1.49 × 10^−7^ (Figure 1e).

### 2.2. Peripheral Blood Cell-Free Transcriptomics

**Figure 2:**
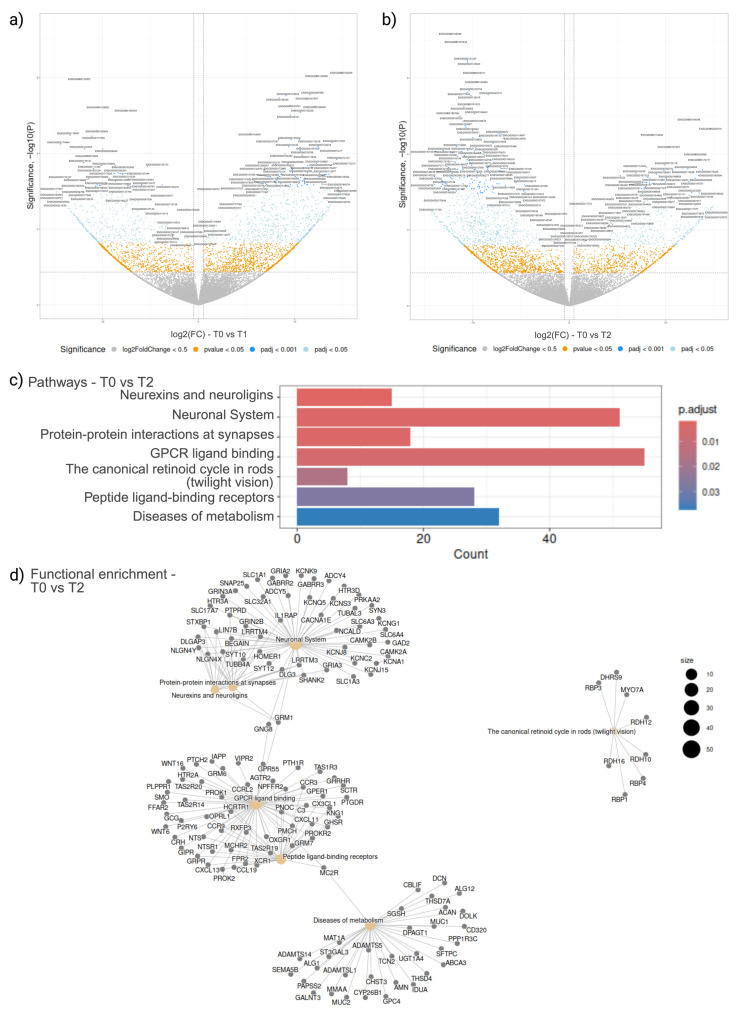
Differential gene expression analysis of cell-free transcriptomics. Volcano plot of DEGs at T1 (**a**) and T2 (**b**), when compared to intake sample at T0, identified with *p*-value < 0.05 and fold change of 1.5 in either direction. Grey: Not significant or under a log2(FC) < 0.5; Yellow: *p*-value < 0.05; Cyan: False discovery rate adjusted *p*-value < 0.05; Blue: False discovery rate adjusted *p*-value < 0.001; (**c**) Bar plot of the significant pathways in when T2 was compared to T0, ranked according to significance; (**d**) Functional enrichment visualisation of pathways found when T2 was compared to T0.

In the comparison between T1 and T0, we identified 1442 DEGs in the cf compartment of peripheral blood, 909 upregulated and 533 downregulated (Figure 2a). The most significant upregulated DEGs identified were PPP1R3C (associated with carbohydrate and glycogen metabolism) and COLEC11 (associated with immunological processes), with adjusted *p*-values of 2.59 × 10^−24^ and 2.11 × 10^−19^, respectively. All cfDEGs, their fold change, and the adjusted *p*-values are listed in Table S1. The most downregulated DEGs at T1 were identified as POSTN (associated with heparin-binding molecular function in cellular adhesion biological processes) and TNFRSF17 (a cell-membrane receptor associated with adaptive immunological processes), with adjusted *p*-values of 2.30 × 10^−16^ and 1.60 × 10^−14^, respectively. While 1105 pathways at T1 were identified (based on *p*-value), such as ‘GPCR ligand binding’ comprising 45 DEGs (*p*-value of 2.92 × 10^−4^), none of the pathways retained significance after adjustment of the *p*-value.

When T2 and T0 were compared, 1676 cfDEGs were identified, 493 upregulated and 1183 downregulated (Figure 2b). The most significant upregulated DEGs at T2 were AGMAT (a hydrolase for arginine metabolism) and ZNF391 (a DNA-binding zinc finger transcription factor), with adjusted *p*-values of 1.31 × 10^−19^ and 2.01 × 10^−14^, respectively. The most significant downregulated T2 DEGs identified were SMARCA1 (associated with transcription regulation) and BRSK1 (a kinase associated with DNA damage and cell cycle), with adjusted *p*-values of 7.69 × 10^−32^ and 7.57 × 10^−27^, respectively. Additionally, seven distinct pathways at T2 were identified with adjusted *p*-values below 0.05 (Figure 2c). Notably, ‘Neurexins and neuroligins’, comprising 15 DEGs, and ‘Neuronal System’, consisting of 51 DEGs, were among the pathways showing significant alterations, with adjusted *p*-values of 2.02 × 10^−3^ and 3.98 × 10^−3^, respectively (Figure 2c). From the functional enrichment plot (Figure 2d), the three most significant pathways identified on Figure 2c, can be observed clustering together.

When the upregulated and downregulated DEGs within the cell-free RNA (cfRNA) dataset were analysed separately, only the 1183 downregulated cfRNA at T2 yielded significant findings, in particular the 13 pathways, including “Neuronal System” with an adjusted *p*-value of 4.71 × 10^−3^, comprising 41 DEGs, identified as most significant (Table S3). Most downregulated T2 cfRNA DEGs were located in the collagen-containing extracellular matrix (GO ID:0062023), while the most significant molecular activity was symporter activity (GO ID:0015293). Biological processes most downregulated at T2 included sensory perception of light stimulus (GO ID:0050953) and visual perception (GO ID:0007601) (Figure S2).

### 2.3. Peripheral Blood Cellular Transcriptomics

**Figure 3:**
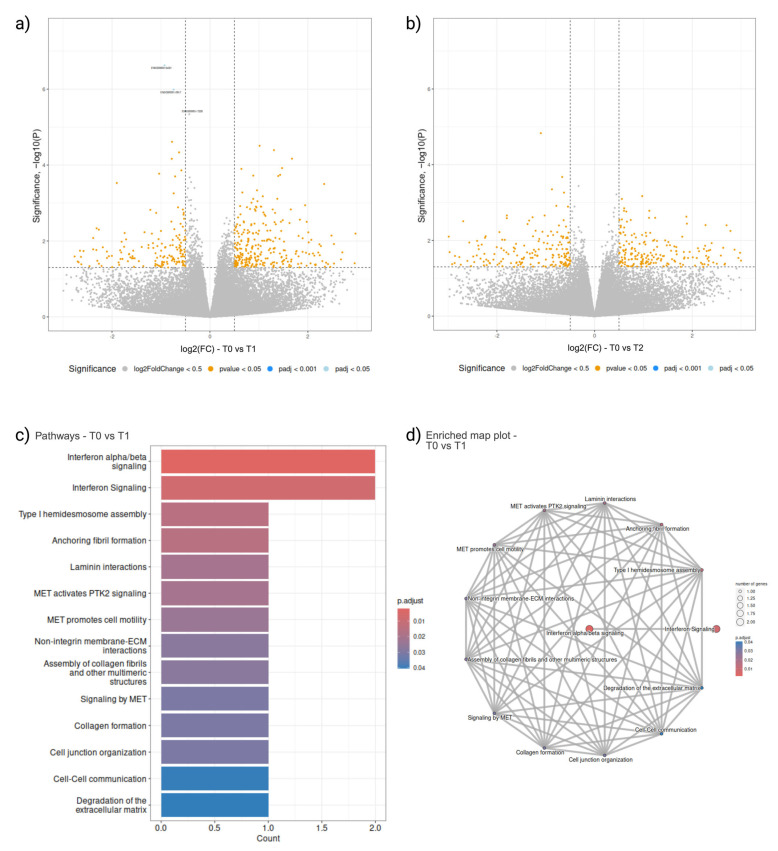
Differential gene expression analysis of cellular transcriptomics. Volcano plot of DEGs at T1 (**a**) and T2 (**b**), when compared to intake sample at T0, identified with *p*-value < 0.05 and fold change of 1.5 in either direction. Grey: Not significant or under a log2(FC) < 0.5; Yellow: *p*-value < 0.05; Cyan: False discovery rate adjusted *p*-value < 0.05; Blue: False discovery rate adjusted *p*-value < 0.001; (**c**) Bar plot of the significant pathways in when T1 was compared to T0, ranked according to significance; (**d**) Enrichment map plot of the DEGs identified in T1 when compared to intake at T0.

The comparison of T1 and T2 time points with T0 yielded few DEGs (Figure 3a,b). When T1 was compared to T0, three downregulated DEGs were identified (Figure 3a). All cellular DEGs, their fold change, and the adjusted *p*-values are listed in Table S1. With an adjusted *p*-value of 1.87 × 10^−6^, heparin-binding, LAMC2, associated with cell adhesion, was identified as the most significant downregulated DEG. RSAD2 and IFIT3, both involved in processes such as anti-viral defence, innate immunity, and general immunity, with adjusted *p*-values of 1.21 × 10^−2^ and 4.15 × 10^−3^, respectively, were also identified as downregulated when at T1 when compared to T0. Enriched pathway analysis identified 14 significant pathways, with ‘Interferon alpha/beta signalling’ and ‘Interferon Signalling’ as the two most significant with adjusted *p*-values of 1.91 × 10^−3^ and 7.37 × 10^−3^, respectively, and associated with RSAD2 and IFIT3 (Figure 3c.) While these two most significant pathways were linked to only each other, dense connections were observed in the other 12 pathways identified (Figure 3d). When T2 was compared to T0, there were no DEGs (Figure 3b), nor pathways identified.

### 2.4. Peripheral Blood Plasma Proteomics

**Figure 4:**
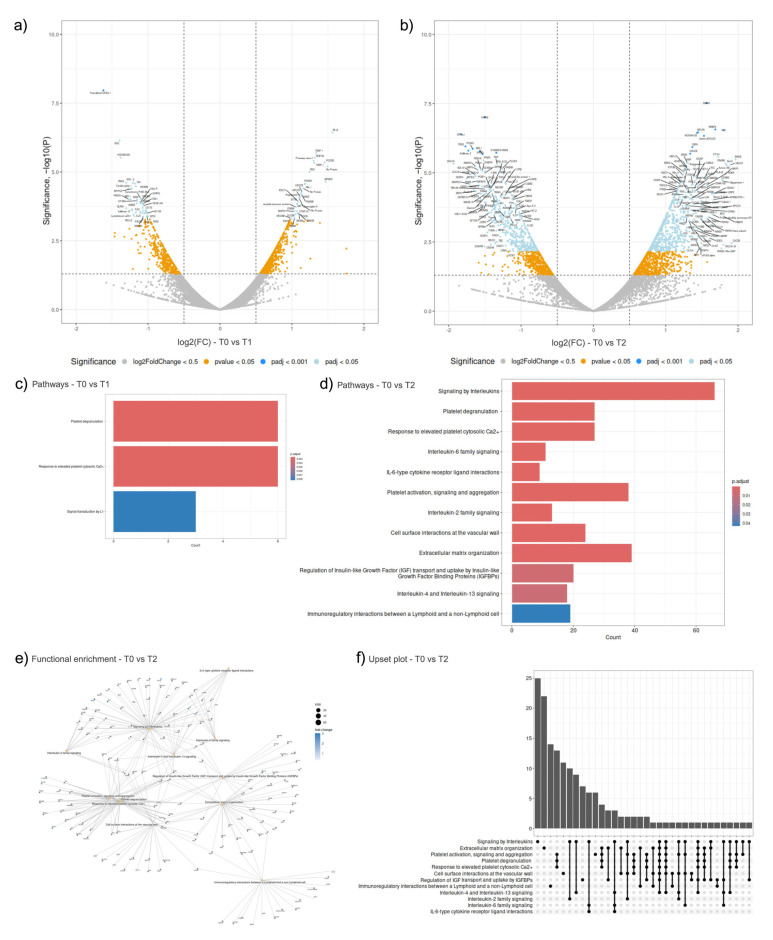
Differential expression analysis of proteomics samples. Volcano plot of DEPs at T1 (**a**) and T2 (**b**), when compared to intake sample at T0, identified with *p*-value < 0.05 and fold change of 1.5 in either direction. Grey: Not significant or under a log2(FC) < 0.5; Yellow: *p*-value < 0.05; Cyan: False discovery rate adjusted *p*-value < 0.05; Blue: False discovery rate adjusted *p*-value < 0.001; (**c**) Bar plot of the significant pathways in when T1 was compared to T0, ranked according to significance; (**d**) Bar plot of the significant pathways in when T2 was compared to T0, ranked according to significance; (**e**) Functional enrichment visualisation of pathways found when T2 was compared to T0; (**f**) Upset plot showing the number of DEPs in the intersection in the pathways identified when T2 was compared to T0. Horizontal rows represent the pathways identified and vertical bars represent the number of DEPs in each intersection.

The comparison of T1 and T2 time points with T0 yielded an abundance of differentially expressed proteins (DEPs) (Figure 4a,b). When T1 was compared to T0, 79 DEPs were identified, 33 upregulated and 46 downregulated (Figure 4a). The most significantly upregulated T1 DEPs were RL12 (RNA-binding ribonucleoprotein) and TIMP-1 (a secreted growth factor and enzyme inhibitor), with adjusted *p*-values of 1.39 × 10^−3^ and 3.85 × 10^−3^, respectively. The most downregulated DEPs were CX3CL-1 (a cytokine associated with cell inflammatory responses) and ITA5 (an integrin receptor), with adjusted *p*-values of 8.23 × 10^−5^ and 1.84 × 10^−3^, respectively, both pathways associated with cell adhesion and host-virus interactions. Pathway analysis of the T1 DEPs identified three pathways, with ‘Platelet degranulation’ and ‘Response to elevated platelet cytosolic Ca2+’ as the most significant, both with adjusted *p*-values of 2.51 × 10^−3^ and comprising six DEPs (Figure 4c).

Among the 46 downregulated T1 DEPs identified, cytokine receptor binding (GO ID:0005126) was the most prominent molecular function, comprising six DEPs and an adjusted *p*-value of 5.22 × 10^−3^ (Figure S3a). There were no significant pathways identified for T1 downregulated DEPs (Table S3). For 33 upregulated T1 DEPs (Figure S3b), the most significant molecular function was associated with endopeptidase inhibitor activity (GO ID:0004866), the most significant biological process affected was the positive regulation of mononuclear cell migration (GO ID:0071677) and the platelet alpha granule (GO ID:0031091) was the most significant cellular compartment these DEPs were localised at (Supplemental Figure S2b). ORA pathway analysis was carried out with the upregulated T1 DEPs and identified eight significant pathways (Figure S3c), listed in Table S3. The most significant pathways were “Platelet degranulation” and “Response to elevated platelet cytosolic Ca2+” both comprising six DEPs, both with adjusted *p*-values of 5.50 × 10^−6^ (Figure S3c).

When T2 was compared to T0, 1031 DEPs were identified, 636 upregulated and 395 downregulated (Figure 4b). The most significantly upregulated T2 DEPs were SMAC (associated with the apoptosis process) and NRBF2 (associated with the autophagy and transcription regulation processes), with adjusted *p*-values of 2.30 × 10^−4^ and 4.40 × 10^−4^, respectively. The most significantly downregulated T2 DEPs were identified as LRFN2 (a leucine-rich protein domain associated with postsynaptic cellular membranes) and DPYL4 (a dihydropyrimidinase-related protein) with adjusted *p*-values of 3.78 × 10^−4^ and 4.40 × 10^−4^, respectively. Pathway analysis of T2 DEPs identified 12 significant pathways, with ‘Signalling by Interleukins’ (comprising 66 DEPs) and ‘Platelet degranulation’ (comprising 27 DEPs) as the most significant with adjusted *p*-values of 9.11 × 10^−8^ and 5.61 × 10^−6^, respectively (Figure 4d). From the functional enrichment plot (Figure 4e) at T2, it can be observed that the five significant interleukin signalling pathways shared multiple common DEPs. Apart from a handful of DEPs, the three haemostasis-related pathways shared common DEPs among one another that were different to the interferon signalling ones (Figure 4e,f).

In the 395 downregulated T2 DEPs, cytokine receptor binding (GO ID:0005126) was again identified as the most significant molecular function with an adjusted *p*-value of 2.29 × 10^−6^ and comprising 23 genes (Figure S4a). The external side of the plasma membrane (GO ID:0009897), comprising 28 genes, was identified as the most significant cellular compartment and the most significant biological process was leukocyte proliferation (GO ID:0070661), with a gene count of 26 (Figure S4a). The most significantly downregulated T2 DEP pathways were “Regulation of Complement cascade” with an adjusted *p*-value of 1.35 × 10^−2^ and “Complement cascade” with an adjusted *p*-value of 1.88 × 10^−2^, both comprised five DEPs (Figure S4b).

The 636 upregulated T2 DEPs were localised most significantly in platelet alpha granules (GO ID:0031091) with an adjusted *p*-value of 1.21 × 10^−10^ and platelet alpha granule lumen (GO ID:0031093) with an adjusted *p*-value of 1.88 × 10^−10^, comprising 21 and 18 DEPs, respectively (Supplemental Figure S3c). The most significant molecular activity identified was growth factor activity (GO ID:0008083) and four significant biological processes associated with peptidyl tyrosine (GO ID:0050731, 0018108, 0018212, 0050730) were identified (Table S2 and Figure S4c). “Platelet degranulation” and “Response to elevated platelet cytosolic Ca2+”, the same two pathways identified at T1, were even more significantly upregulated at T2, both with 24 DEPs and adjusted *p*-values of 1.24 × 10^−7^ and 1.42 × 10^−7^, respectively (Figure S4d).

### 2.5. Integration

Data Integration Analysis for Biomarker discovery using the Latent cOmponent (DIABLO) identified markers that exhibited the highest correlation across our multi-omics datasets (Figure S5) and then singled out a minimal number of markers with the greatest predictive value (Table S4). These selected markers were then used to construct molecular signatures for distinguishing between different time points. Fine-tuning the performance of the final DIABLO model revealed that employing two components (Figure S6) achieved the lowest classification error rate, taking into account prediction distance, and enabled the selection of variables across the datasets derived from disparate types of biological measurements (Supplemental Material). Component one comprised 15 cfRNAs, 10 cellular RNAs, 20 proteins, six metabolites and 10 epigenetic methylation sites, while component two comprised 10 cfRNAs, five cellular RNAs, 10 proteins, nine metabolites and 20 epigenetics methylation sites (Table S4). Within the six participants, component one from all omics datasets is highly correlated (Figure S7), with the lowest correlation between mRNA and proteomics at 0.81 and the highest correlation between mRNA and metabolomics at 0.95. For component two, the highest correlation was between cfRNA and epigenetics at 0.97, while the lowest was between proteomics and metabolomics at 0.7 (Figure S8).

**Figure 5:**
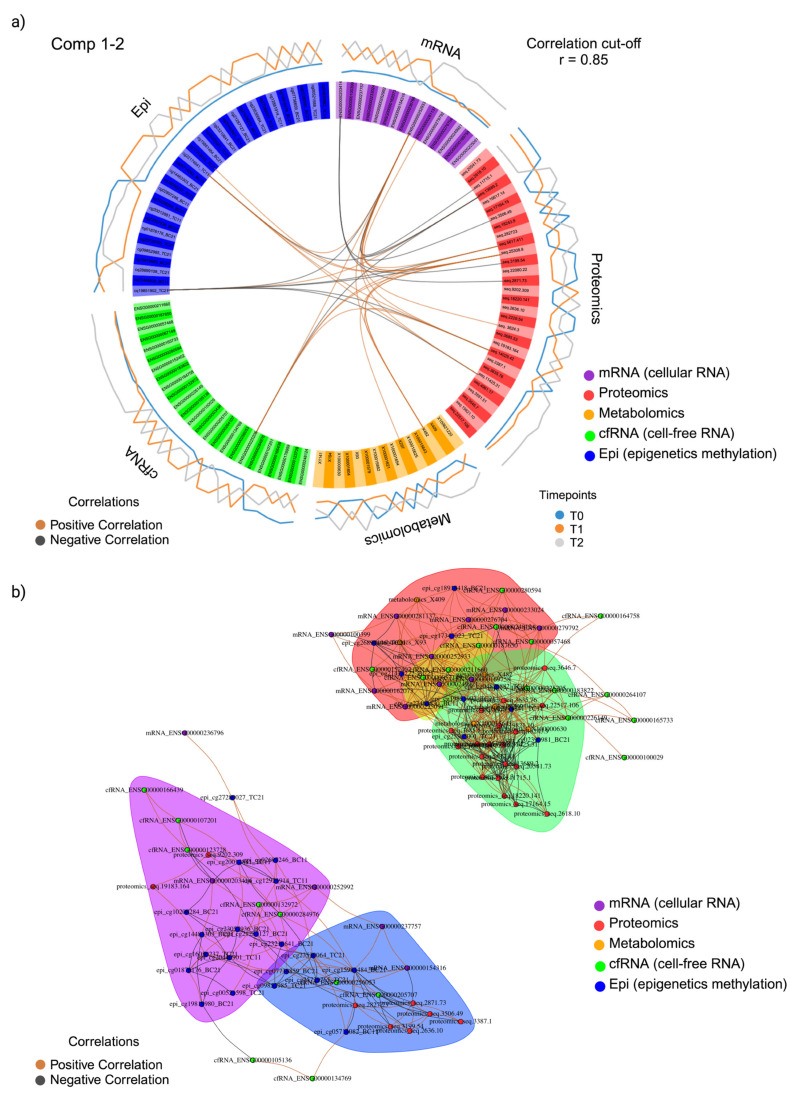
A multi-omics expression signature has been established by examining the correlations among selected features across multi-omics datasets over two components. (**a**) A DIABLO circos plot was generated using a correlation cut-off set above 0.85. The plot encompasses multiple omics datasets, which are distinguished by colour: cellular mRNA (purple), proteomics (red), metabolomics (yellow), cfRNA (green), and epigenetics (blue), organized in respective quadrants. Within the plot, the internal lines connecting the selected variables are indicative of positive (brown) or negative (black) correlations. The external lines represent the expression activity of the corresponding variable at different time points, where T0 is denoted in blue, T1 in orange, and T2 in grey; (**b**) A relevance network has been constructed to illustrate the associations among selected features on components 1 and 2. This network is based on a pairwise similarity matrix derived from multiple omics datasets, with each dataset node represented by colour: cellular mRNA (purple), proteomics (red), metabolomics (yellow), cfRNA (green), and epigenetics (blue). Within the network, the colour of the edges serves as a visual indicator of the relationships between the nodes. Positive correlations are represented by orange edges, while negative correlations are denoted by black edges.

Visualising correlations, both within and between omics datasets using a cut-off set at 0.85, the circos plot displays a blend of positive and negative correlations among the selected features across components one and two (Figure 5a). Notably, these correlations span different omics datasets, with the majority of these associations being observed with the proteomics variables. The pseudogene ENSG00000203414, exhibiting a variance value of −0.89 (Table S4), displayed a negative correlation with three selected proteins: seq.3506.49 (lymphotoxin LTA/LTB), seq. 3199.54 (Kallikrein-12), and seq. 2871.73 (DNA repair protein RAD51) (Figure 5a). Using a pair-wise similarity matrix, a relevance network was constructed to offer insights into the interactions and connections between the selected features across the different omics datasets using a predefined cut-off threshold of 0.75. The features selected for the biomarker panel constituted five interconnected clusters on the network (Figure 5b). The Group 4 cluster, visualised as a green cluster in Figure 5b, contained the most features, comprising 32 variables, namely one mRNA, 19 proteins, four metabolites, three cfRNAs, and five methylation sites. Pathway analysis of the Group 4 cluster identified three pathways associated with the TCA cycle, namely ‘pyruvate metabolism’, ‘pyruvate metabolism and the citric acid (TCA) cycle’ and ‘the citric acid (TCA) cycle and respiratory electron transport’ in order of significance based on adjusted *p* values.

**Figure 6:**
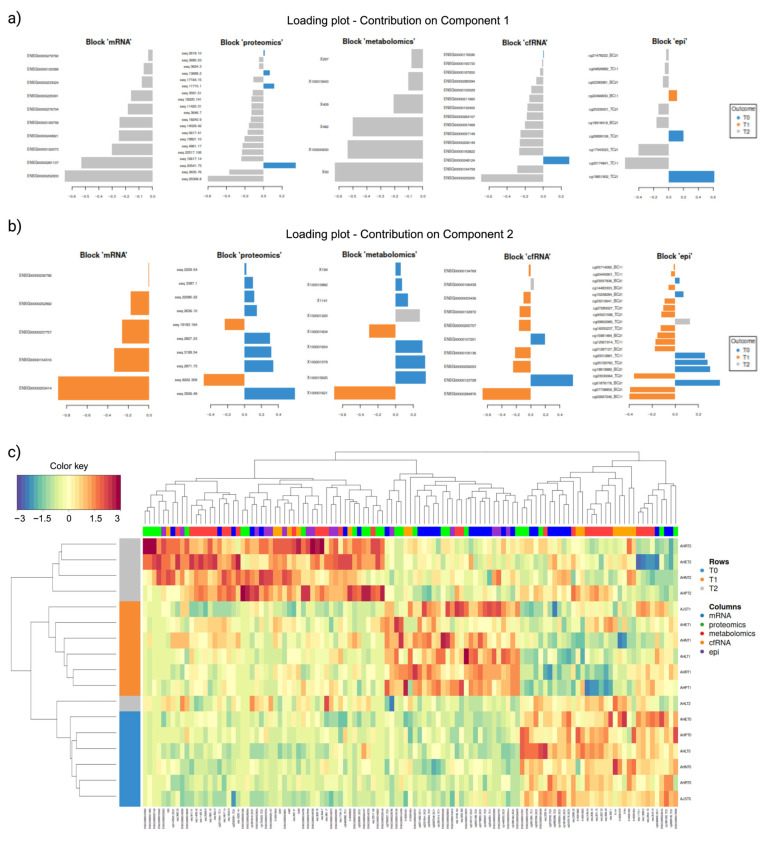
(**a**) Loading plot of DIABLO selected discriminant features on component one; (**b**) Loading plot of DIABLO selected discriminant features on component two. For a and b, from left to right, the loading plot shows the most variable molecular features (y-axis) across time points in mRNA, proteomics, metabolomics, cfRNA, and epigenetics datasets, with the length of the bar representing the contribution weight; (**c**) Hierarchically clustered image map for DIABLO-selected variables, where the rows represent the time points, T0 (blue), T1 (orange), and T2 (grey), and the columns represent the dataset the selected feature belongs to cellular mRNA (blue), proteomics (green), metabolomics (red), cfRNA (orange), and epigenetics (purple).

The loading plots reveal which variables contribute more for each omic type in each component, highlighting distinctive patterns (Figure 6a,b). The most significant molecular variances across all omics datasets when comparing the three time points (T0, T1, and T2) reveal that molecules at T2, featuring negative coefficients, contribute the most discriminative features in component one, effectively segregating T2 from the other time points (Figure 6a). In component two, discriminant features are evident across datasets, with molecular variances at T1 displaying negative coefficients, while the molecular variances at T0 exhibit positive coefficients, effectively distinguishing T0 from T1 (Figure 6b). For a comprehensive list of the molecular features identified by DIABLO and featured in both the loading plots and clustered image maps see Table S4.

Hierarchical clustering employing the Euclidean method was conducted for component one features, revealing distinct molecular patterns associated with each timepoint across datasets (Figure 6c). Within these patterns, a consistent set of discriminant features was observed, reflecting a similar expression profile across time points. In the clustered image heatmap (Figure 6c), all samples, except for participant AHL at T2 (AHLT2), exhibited clustering based on their respective time points. This clustering was indicative of the presence of overexpression signatures in the majority of T2 samples (Figure 6c). Participant AHL at T2 failed to exhibit the same overexpression signatures observed in the other T2 samples. Instead, this particular sample loosely clustered with other T0 samples (Figure 6c). It is of note that serological analysis of participant AHL indicated seroconversion to *Coxiella burnetii*, the causative agent of Q-fever, at T2 (Table S1).

**Figure 7:**
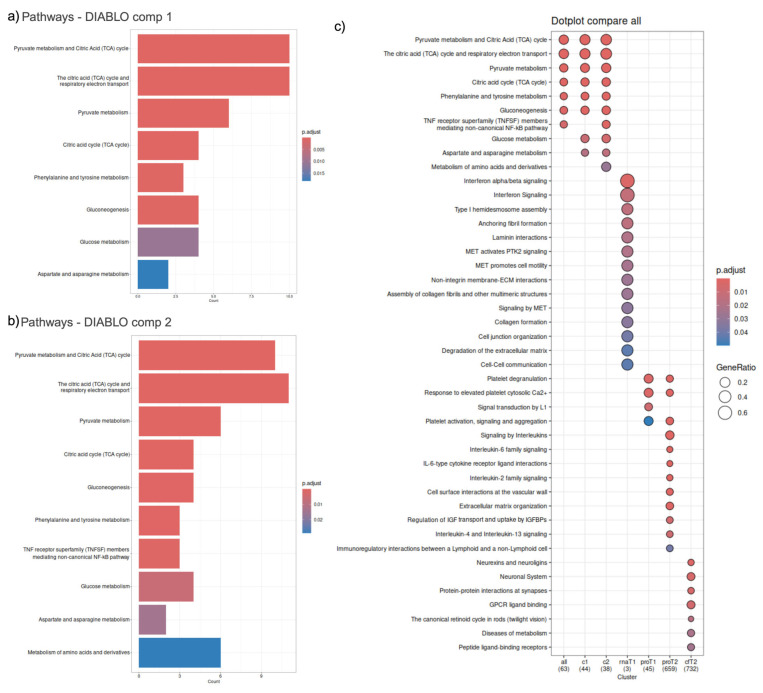
Pathway analysis of DIABLO selected features. (**a**) DIABLO component 1 pathways, ranked according to significance; (**b**) DIABLO component 2 pathways, ranked according to significance; (**c**) Comparison of DIABLO pathways with pathways identified in single omics analysis.

Pathway analysis of the DIABLO selected features in component one identified eight pathways, with four TCA cycle associated pathways identified as the most significant—namely ‘pyruvate metabolism and citric acid (TCA) cycle’ (comprising 10 features), ‘citric acid (TCA) cycle and respiratory electron transport’ (comprising 10 features), ‘pyruvate metabolism’ (comprising six features), and ‘citric acid cycle (TCA cycle)’ (comprising four features), with adjusted *p*-values of 1.37 × 10^−12^, 9.79 × 10^−8^, 9.79 × 10^−8^, and 5.73 × 10^−5^, respectively. (Figure 7a). Component two of DIABLO selected features identified the same four TCA cycle-associated pathways as the most significant in the same order (Figure 7b), comprising 10, 11, 6, and 4 features, with adjusted *p*-values of 3.13 × 10^−13^, 1.08 × 10^−9^, 4.57 × 10^−8^, and 3.70 × 10^−5^, respectively (Table S4).

The pathways derived from DIABLO selected features were compared to the pathways identified with single omics analysis (Figure 7c), revealing different profiles of biological pathways. DIABLO component one, made up of the most predictive features of T2, comprised 44 selected features. In comparison, 659 proteins and 732 cfRNA were discriminative of T2 in single omics. DIABLO component two, represents the most distinctive features that can discriminate between T1 and T2, comprising 38 features. In contrast, three cellular RNAs and 45 proteins were discriminative of T1.

### 2.6. Correlational Analysis of Local (Solid Biopsy) and Systemic (Liquid Biopsy) Signals

**Figure 8:**
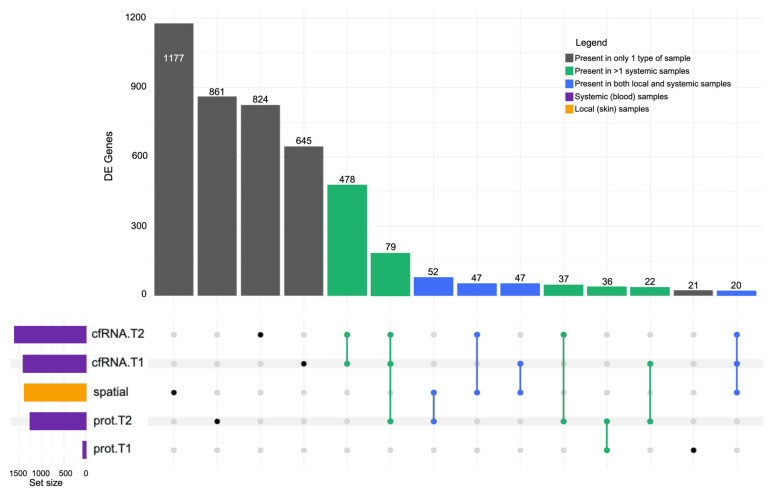
Upset plot comparing the DEGs from local skin (horizontal orange bar) samples set to systemic blood (horizontal purple bars) sample sets, comprising differentially expressed cell-free RNA and proteomics derived from peripheral blood drawn at three time points (T0, T1, and T2). DEGs present only in one type of sample were presented in grey, in more than one systemic sample type in green and in both local and systemic sample types in blue.

The data from local events (skin tissue) [7] and peripheral blood samples were compared and contrasted to investigate the intersections between local and systemic signals. Out of the 1380 DEGs identified in the skin, 1177 (85%) occurred uniquely in the skin (Figure 8). Similarly, cfRNA T1(645 DEGs), cfRNAT2 (824 DEGs), proteomics T1(21 DEGs), and proteomics T2 (861 DEGs) sample sets had DEG/DEPs that were not common in the other (tissue) data set (Figure 8). However, local (tissue) samples shared several common DE molecules with systemic (liquid) samples (blue columns), namely proteomics T2 (52 DEGs), cfRNA T1(47 DEGs), and cfRNA T2 (47 DEGs).

The extracellular matrix (ECM) organisation (R-HSA-1474244) pathway experienced an upregulation in the tick-bitten skin on intake at T0 when compared to the contralateral control, comprising 29 DEGs (Figure 1e). Four proteins at T1 and 27 proteins at T2, that were part of the ECM organisation pathway, were upregulated in comparison to T0. Interestingly, 31 downregulated cfRNA DEGs, that were part of the ECM organisation pathway, were observed at T2. The other consistently identified pathways across time points were “Platelet degranulation” (R-HSA-114608) and “Response to elevated platelet cytosolic Ca2+” (R-HSA-76005) which were observed to comprise 16 upregulated DEGs locally in the skin (Figure 1e), followed by 6 DEPs at T1 (Figure S3c), and 24 DEPs at T2 (Figure S4d) when compared to T0.

Enrichment analysis carried out with GO on the DEGs showed that the heparin-binding (GO ID:0008201) molecular function exhibited an initial upregulation locally (Figure 1d) with 18 DEGs and concurrently within the first-week post-intake, displaying an upregulation in proteomics at T1 (Figure S3b) with a further increase of three DEGs. However, its expression was subsequently downregulated in both T2 samples when compared to T0, namely proteomics (Figure S3a) with 11 DEPs downregulated and cfRNA (Figure S2) with 18 DEGs. Furthermore, 31 DEGs were downregulated in the skin (Figure 1b) which was part of the cytokine receptor binding (GO ID:0005126) molecular function, which experienced a further downregulation of 6 proteins at T1 (Figure S3a). For the cytokine receptor binding function at T2, 23 proteins were downregulated (Figure S4a) while a set of 26 proteins from the same molecular function were upregulated (Figure S4c).

Gene ontology also pinpointed the cellular compartment, collagen-containing extracellular matrix (GO ID:0062023), across data sets with 41 upregulated DEGs from the skin identified (Figure 1d), followed by an additional upregulation of 5 plasma proteins at T1. Localised at the collagen-containing extracellular matrix at T2, were 32 upregulated plasma proteins (Figure S4c) and 48 downregulated cfDEGs (Figure S2). Biological processes observed across sample types include upregulated processes such as negative regulation of peptidase activity (GO ID:0010466), negative regulation of endopeptidase activity (GO ID:0010951), and regulation of peptidase activity (GO ID:0052547) and 15 DEGs, respectively identified in the skin (Table S2). These three biological processes identified went on to experience further upregulation in DEPs at T1 and T2.

## 3. Discussion

The diverse symptomology experienced by humans following a tick bite may impede the timely diagnosis of TBDs, and this has consequently hindered insight into the early underlying pathophysiological mechanisms [11,12]. This study aimed to address this knowledge gap by correlating local with systemic signals. Spatial transcriptomics of paired skin biopsies [7] enabled the identification of local tick-bite signals on the day of enrolment. As we show here, concurrent (i.e., at the time of skin biopsy) and subsequent, longitudinal sampling of peripheral blood after tick bite allowed systemic signals to be observed and compared to the time of enrolment, at one week, and at three months, offering a glimpse into the trajectory of perturbations of the local biological processes.

Our previous investigation [7] revealed an abundance of local skin signatures that, as expected, exhibited a larger range of DEGs and enriched pathways than systemic blood signatures described in this study. This is consistent with comparable research where tissue biopsies demonstrated superior detection rates of clinically significant perturbations compared to liquid biopsies [13,14,15]. Nonetheless, liquid biopsies retain their value in scenarios where tissue testing is challenging, such as in cases necessitating sequential sampling [13,14,15]. Comparisons between tick-bitten and contralateral control skin collected upon enrolment (T0) revealed an upregulation in DEGs that are part of extracellular matrix (ECM) organisation and platelet degranulation pathways [7]. Importantly, as we demonstrated here, these pathways also share a pattern of increased activity in the systemic (blood) proteomics dataset, at one week and three months post tick bite. These signals, in both the local and systemic data, could reflect the active involvement of the platelet degranulation pathway in haemostasis and the ECM organisation pathway in the repair of the cutaneous wound inflicted by tick bites [16].

In the proteomics dataset, the ECM organisation pathway demonstrated a marginal increase in activity from T0 to T2. Conversely, the cfRNA data did not detect any perturbations in the ECM organisation pathway between T0 and T1 but exhibited a marked downregulation of ECM organisation pathway activity at T2, potentially indicating the conclusion of the wound repair process as the participants approached the three-month mark post tick bite. In cases of unresolved tick-associated conditions, it is suggested that these signals may align with dermatological lesions in these patients and may persist [17,18,19]. Similarly, changes to haemostasis post tick bite, as demonstrated in this study, can also lead to coagulopathies like thrombocytopenia from the over-consumption of coagulation mediators such as platelets [20,21]. Therefore, monitoring of these signals post-treatment could provide prognostic insight into the persistence or resolution of symptoms.

Fluctuating expression patterns of gene ontologies across different types of samples (local and systemic) and over multiple time points were demonstrated in heparin binding and cytokine receptor binding molecular functions. A tick bite not only initiates haemostasis and antimicrobial activities via heparin-binding activities but also introduces pathogen-associated molecular patterns from the pathogens inoculated and the release of damage-associated molecular patterns by injured host cells, both of which are recognised and can bind to host pattern recognition receptors [1,22]. The engagement of these receptors can trigger a cascade of molecular mechanisms within the host, including the activation of inflammasomes and fluctuating cytokine activities [1,23]. These fluctuations in cytokine binding activities often reflect the dynamic and complex interplay of the human response following a tick bite [23,24].

This pilot study suggests that shared signals between local and systemic datasets in tick-bitten individuals exist and, more importantly, can be measured and tracked through serial peripheral blood samples. To further validate the potential of this approach as being capable of detecting local molecular perturbations discernible at a systemic level for TBDs, and to expand this methodology to a broader range of applications, including other vector-borne diseases [5,6], it is now reasonable to design larger follow-up studies around this proof-of-concept platform.

While the experimental design translates into a robust workflow, the sample size in this study is a limitation. Furthermore, power calculations were not undertaken due to the absence of a clinical endpoint or phenotype. Other limitations include the sex ratio of the cohort and the relatively short duration of tick attachment in many cases. The cfRNA investigation conducted here is a relatively new technique [4,25,26]. With strict adherence to transcriptomics analysis guidelines, an abundance of cfRNA DEGs was detected at all follow-up time points when compared to intake at T0. However, downstream analysis of the identified cfRNA DEGs by querying publicly available, curated databases, yielded a paucity of results in terms of enriched pathways or gene ontogeny. This limitation may stem from the recency of gene annotations within these databases and the untargeted list of input genes utilised in this analysis, primarily due to the lack of prior knowledge concerning mRNA associated with TBDs [25,26,27]. Future iterations of these databases can potentially be employed to analyse historical samples, such as the one generated from this study, to derive additional insights.

Despite these limitations, the results of this study represent the success of a ‘proof-of-concept technical pilot’ that attests to the feasibility of this liquid biopsy approach. We emphasise that this pilot was not intended for biological interpretations of human tick bite cases. Yet, despite the small sample size, these data demonstrate that identifying local molecular signals common to local tissue and systemic alterations in blood is possible. Conceptually, this study shows that systemic signals, particularly liquid biopsy signals derived from peripheral blood can capture local skin-based tick-associated signals. This provides a novel avenue to detect and investigate tick-associated illnesses that were undetectable with current methods. In the long term, these findings will also need to be compared to the effects of other bites (for example, bites from fleas, spiders, and mosquitoes) in contrast to purely mechanical damage to the skin.

## 4. Materials and Methods

### 4.1. Overview

**Figure 9:**
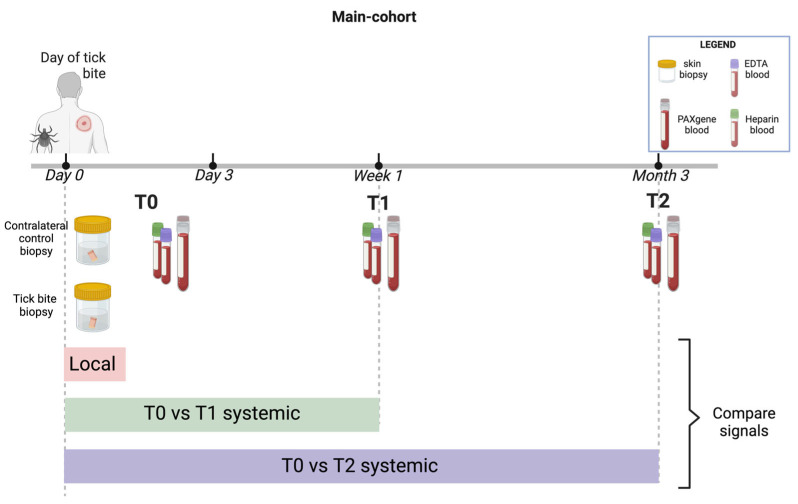
Tick-bitten participant sample collection schedule. On enrolment (T0 = within 72 h (up to Day 3) from tick bite), two skin biopsies and a set of blood samples were collected from study participants. On subsequent follow-up time points (T1 = one-week post enrolment and T2 = three months post enrolment), only venous blood samples were collected. Created with BioRender.com.

The experimental study design, detailed in Figure 9, included sample processing that adhered to guidelines set by the Murdoch University Human Research Ethics Committee (permit 2019/124) as part of a broader research effort investigating human tick-associated diseases in Australia [28]. Written informed consent was obtained from all participants before enrolment and collection of their samples.

### 4.2. Data Selection Criteria and Extraction

Each participant contributed a set of samples, including the tick responsible for the bite, two skin biopsies (one extracted from the tick bite site, the other from the contralateral (control) site), whole blood samples collected into PAXgene^®^ Blood RNA tubes (PreAnalytiX GmbH; Hombrechtikon, Switzerland), EDTA, and lithium heparin tubes (Figure 9). Inclusion criteria comprised participants (a) who were enrolled and sampled within 72 h of tick bite and (b) were able to provide samples for at least one of the cohort aims (local or systemic studies). Exclusion criteria include (a) children under 18 years of age; (b) current pregnancy; (c) previous diagnosis of chronic fatigue syndrome, fibromyalgia, myalgic encephalomyelitis, Lyme disease (LD) or chronic “LD-like” illness); and patients with coagulopathy or on anticoagulant therapy (except aspirin) [28]. All participants except one (AHP, who declined consent for the contralateral control biopsy) provided the full sets of samples. As such, participant AHP was omitted from the spatial analysis due to a lack of a control sample but was included in the whole blood systemic analysis.

### 4.3. Participant Details

A set of clinical information pertaining to the selected participants was extracted, encompassing both participant metadata and processing metadata, including quality assurance and control, processing, and normalisation details (Table S1).

### 4.4. Spatial Transcriptomics

Spatial transcriptomics data for each participant (from Lee et al., 2024) were downloaded for analysis using R v4.3.1 (R Foundation for Statistical Computing, Vienna, Austria) [29]/Bioconductor packages v3.18.0 and NanoString-validated packages (NanoString Technologies, Inc., Seattle, WA, USA) for R, namely GeoMxWorkflows (v1.6.0) [30] and GeoMxTools (v 3.5.0) [31,32] as previously described [7]. DEGs were ascertained by employing a linear mixed-effect model (LMM) with a random slope, where genes with an observed fold change of 1.5 in either direction and adjusted *p*-value of 0.05 (Benjamini-Hochberg).

### 4.5. Cell-Free Transcriptomics

Whole blood, collected via venepuncture directly into EDTA Vacutainer tubes (Becton Dickinson, NJ, USA), was centrifuged at 1000× *g* at room temperature for 15 min to obtain platelet-rich plasma (PRP). The cell-depleted PRP was meticulously collected via an RNase-free micropipette and was centrifuged at 2500× *g* at room temperature for 15 min to obtain platelet-poor plasma (PPP) and stored at −80 °C until further usage for cfRNA extraction. Haemolysis scoring on the PRP was measured at 414nm using the NanoDrop 2000 (Thermo Fisher Scientific; Waltham, MA, USA) and cfRNA was extracted using the Qiagen miRNeasy Serum/Plasma Advanced Kit (Qiagen, Hilden, Germany) in accordance with manufacturer’s instructions (Figure S1). Extracted cfRNA was checked for genomic DNA (gDNA) contamination with SsoAdvanced Universal SYBR Green Supermix (BioRad, Hercules, CA, USA) via qPCR on C1000 TouchTM Thermal Cycler (Bio-Rad Laboratories, Inc., Hercules, CA, USA) and CFX Real-Time PCR Detection Systems (Bio-Rad Laboratories, Inc., Hercules, CA, USA), and RNA quality was assessed via electrophoresis using the Eukaryote Total RNA Pico assay on the 2100 bioanalyzer (Agilent Technologies Inc., Santa Clara, CA, USA). The Illumina RNA Prep with Enrichment (L) Tagmentation kit (Illumina; San Diego, CA, USA) was used to construct a library with index adapters from IDT for Illumina DNA/RNA Index Set A (Illumina; San Diego, CA, USA) and exome panel with enrichment oligos (Illumina; San Diego, CA, USA). High output (50–60 Gb) RNA sequencing of 75 bp paired-end reads was carried out on the Illumina NextSeq 500/550 platform (Illumina; San Diego, CA, USA) at Norgen BioTek Corp (Figure S1). Initial quality control checks were conducted using FastQC v0.12.0 (Babraham Institute, Cambridge, UK) and MultiQC v1.13 (Seqera Labs, Barcelona, Spain) [33]. To facilitate transcript alignment, STAR v2.7.10b (Cold Spring Harbor Laboratory, Cold Spring Harbor, NY, USA) [34] was used to create a reference genome utilising the hg38 human genome (Ensembl GRCh38.86) [35] (Figure S1). Subsequently, the untrimmed FASTQ sequences, including adapters and indexes, were aligned to hg38 transcripts using STAR (Figure S1). The resulting binary alignment map (BAM) files were indexed using Samtools 1.16.1 (Genome Research Ltd., Hinxton, UK), yielding sequence alignment map (SAM) files [36]. Read counts were then computed from the SAM files using HTseq v2.0.2 [37], leading to the generation of raw RNA count files.

Differential gene expression analysis was conducted using the DESeq2 version 1.38.3 [38] package within R v4.3.1 [29], Bioconductor packages v3.18.0 (Figure S1). Genes with a normalised count of under five in less than three samples as well as globin genes (specifically “ENSG00000206172”, “ENSG00000188536”, and “ENSG00000244734”) were bioinformatically excluded [39]. DEGs were defined as genes exhibiting a fold change of at least 1.5 in either direction using the Wald test and an adjusted *p*-value of 0.05 (Benjamini-Hochberg). Pathway analysis was carried out using ReactomePA (v1.44.0) [40].

### 4.6. Cellular Transcriptomics

Whole blood samples collected via venepuncture directly into PAXgene blood RNA tubes (PreAnalytiX GmbH; Hombrechtikon, Switzerland) were inverted 10 times immediately after collection and left to incubate at room temperature for at least 2 h before being stored at −80 °C until RNA extraction. Cellular RNA was manually extracted from whole blood collected in PAXgene blood RNA tubes using a PAXgene blood miRNA kit (PreAnalytiX GmbH; Hombrechtikon, Switzerland) following the manufacturer’s instructions (Figure S1). Total cellular RNA was quantified on the NanoDrop 2000 (Thermo Fisher Scientific; Waltham, MA, USA) and the RNA quality was assessed via electrophoresis using the LabChip GX nucleic acid analyser (PerkinElmer; Waltham, MA, USA). The pure RNA samples obtained were used to generate libraries using the Illumina stranded mRNA Library Prep (Illumina; San Diego, CA, USA), perform qPCR quantification for further quality control and perform RNA sequencing of 150 bp paired-end reads at a sequencing depth of 50 million on the Illumina NovaSeq 6000 platform (Illumina; San Diego, CA, USA) at Australian Genome Research Facility. The raw FASTQ data were transferred via RSYNC into a Linux virtual machine for pre-processing. FASTQ sequence reads with more than one file were concatenated and initial quality control was performed using FastQC v0.12.0 and MultiQC v1.13 [33]. STAR v2.7.10b was used to generate a reference genome with Ensembl GRCh38.86 [35] and align the untrimmed FASTQ sequences (inclusive of adapters and indexes) to hg38 transcripts. The BAM files generated by STAR were indexed by Samtools 1.16.1 and SAM files [36]. HTseq v2.0.2 [37] was used to perform read counts on the SAM files and generate raw RNA count files. Differential gene expression analysis was performed as described above.

### 4.7. Plasma Proteomics

Whole blood samples collected via venepuncture directly into lithium heparin Vacutainer^®^ tubes (Becton Dickinson, Franklin Lakes, NJ, USA) were centrifuged at 1000× *g* at room temperature for 10 min. The plasma collected via a sterile micropipette (while ensuring the red cells remain untouched) was aliquoted and stored at −80 °C until further analysis. The plasma samples were then analysed in one batch on the SomaScan^®^ platform by SomaLogic Inc. (Boulder, CO, USA) (Figure S1). Single-stranded DNA aptamers, known as SOMAmers^®^, were used to target approximately 7000 proteins in each plasma sample and the measurement of these SOMAmers^®^ was performed using microarray for relative protein quantification. The raw data was normalised against hybridisation controls and pooled calibrator replicates and analysis was carried out using R v4.3.1 [29] with the Differential expression testing with linear mixed models for repeated measures (Dream) [41] using a LMM. DEPs were determined as proteins with an observed fold change of 1.5 in either direction with an adjusted *p*-value of 0.05 (Benjamini-Hochberg).

### 4.8. Plasma Metabolomics

Whole blood, collected via venepuncture directly into lithium heparin Vacutainer^®^ tubes (Becton Dickinson, Franklin Lakes, NJ, USA) was centrifuged at 1000× *g* at room temperature for 10 min. The plasma layer free of blood cells was collected as described above, aliquoted, and stored at −80 °C awaiting further analysis. The plasma samples were then analysed in one batch on the untargeted metabolomics platform at Metabolon Inc. (Durham, NC, USA) (Figure S1). The extraction of metabolites from the plasma samples was carried out via methanol precipitation on the automated MicroLab STAR^®^ system (Hamilton Company, Reno, NV, USA) and divided into five aliquots to be run on four separate methods. The HD4 Waters ACQUITY method of ultra-performance liquid chromatography (UPLC) (Waters Corporation, Milford, MA, USA) was carried out in tandem with a Q-Exactive mass spectrometry (MS/MS) (Thermo Fisher Scientific, Waltham, MA, USA) with a heated electrospray ionization (HESI) coupled to an Orbitrap mass analyser (Thermo Fisher Scientific, Waltham, MA, USA) set at 35,000 mass resolution to identify and provide a relative quantification of approximately 5400 metabolites in all methods. Aliquot one was run on a reverse-phase (RP) UPLC-MS/MS coupled to a positive ion mode HESI optimised for hydrophilic compounds, aliquot two was run on RP/UPLC-MS/MS coupled to a positive ion mode HESI optimised for hydrophobic compounds, aliquot three was run on RP/UPLC-MS/MS coupled to a negative ion mode HESI, aliquot four was run on HILIC/UPLC-MS/MS coupled to a negative ion mode HESI and aliquot five was reserved as a spare. The raw data was batch-normalised, imputed and log-transformed for analysis on R v4.3.1 [29]. Metabolites were considered differentially expressed based on an observed change of 1.5-fold in either direction using a linear model with an adjusted *p*-value of 0.05 (Benjamini-Hochberg).

### 4.9. Whole Blood Cell DNA Methylation

Whole blood, collected via venepuncture directly into lithium heparin Vacutainer^®^ tubes (Becton Dickinson, Franklin Lakes, NJ, USA), was centrifuged at 1000× *g* at room temperature for 10 min. The plasma was removed using a sterile micropipette, leaving the packed red cell pellet and the white cell buffy coat untouched. The cell pellet was then resuspended and stored at −80 °C until gDNA extraction. The gDNA was extracted using the automated Chemagic™ 360 instrument (Revvity, Waltham, Massachusetts, USA) and Chemagic™ DNA Blood 400 Kit H96 (cat# CMG-1901, Revvity, Waltham, Massachusetts, USA), in accordance with manufacturer instructions (Figure S1). The concentrations of the purified gDNA samples were then quantified using the Qubit™ dsDNA High Sensitivity kit and NanoDrop 2000 (both from Thermo Fisher Scientific; Waltham, MA, USA) as part of a quality control step. Methylation assay was conducted using the Illumina MethylationEPIC v2.0 BeadChip kit (Illumina; San Diego, CA, USA) to interrogate the methylation of more than 935,000 CpG sites of the human methylome. This assay was performed by the Australian Genome Research Facility in Melbourne. Minfi v3.17 [42] was used to pre-process the raw .iDAT files, incorporating control probes to assess sample quality. This process included between-array normalisation, evaluation of the P-detection call rate and the removal of probes exhibiting off-target effects. Analysis was carried out using R v4.3.1 [29] with Dream [41] using a LMM. Differentially methylated genes (DMGs) were determined with an observed fold change of 1.5 in either direction with an adjusted *p*-value of 0.05 (Benjamini–Hochberg).

### 4.10. Data Integration

The multi-variate DIABLO approach, a module in the MixOmics R package [43], was used to provide insight into the progression of tick bites from five highly-dimensional, discrete datasets, namely, cellular transcriptomics, cell-free transcriptomics, proteomics, metabolomics, and whole blood DNA methylation. The data sets were processed in R v4.3.1 [29] to remove zero values and low counts (defined as cumulative methylation under 0.1 and a cumulative count of under 10 across all samples for all other omics). A multinomial generalised linear model was used to perform LASSO regression for feature selection of the most variable features. The final processed datasets were normalised (z-score normalisation), centred and log-transformed to obtain input matrices for integration [44,45]. Sparse projection to latent structures-determinant analysis (sPLS-DA) [44] was used to perform the integration of input matrices and build components with DIABLO. The time points (T0, T1, and T2) were set as response variables, and DIABLO was used to maximise the covariance between the input matrices and response variables to identify the main determinants to differentiate time points. A basic DIABLO model was constructed with a design matrix with a correlation of 0.1 to identify correlated variables across data sets for 10 components. Hyperparametric performance tuning was carried out to ascertain the optimal number of components (based on the classification error rates after 5-fold of 20-*n* repeats) and features (based on 5-fold of 2-*n* repeats cross-validation) to run the final DIABLO model. The performance of the final DIABLO model was assessed with a diagnostic plot for each relevant component and the correlation as well as clustering quality of the selected features from each dataset was assessed with DIABLO (Supplemental Materials). Pathway analysis on the features selected by the DIABLO model was performed by ReactomePA (v1.44.0) [40] and the *p*-values adjusted with the Benjamini–Hochberg method [46].

### 4.11. Correlation Analysis and Visualisation

All differentially expressed proteins or transcripts were mapped to common gene identifiers using biomaRt (v 2.58.0) [47] and the genome-wide human annotation org.Hs.eg.db (v3.18.0) on Bioconductor [48]. Enrichment analysis of DEGs was carried out with gene ontogeny (GO) [49,50] to categorise subontologies of biological domains using clusterProfiler (v.4.10.0) [51] and enrichplot (v. 1.22.0) [52]. Over-representation analysis (ORA) to identify pathways was carried out using ReactomePA (v1.44.0) [40].

## Data Availability

All data are publicly available. The transcriptomics data presented in this publication were submitted to the NCBI Gene Expression Omnibus under accession number GSE286962. All other datasets including cfRNA, proteomics, metabolomics, and epigenetics datasets presented in this publication have been archived on FigShare under DOI: https://doi.org/10.6084/m9.figshare.25772382.v1.

## Supplementary Materials

The following supporting information can be downloaded at: https://www.mdpi.com/article/10.3390/ijms26041520/s1.
