# Supplementary material for: From Local to Systemic: The Journey of Tick Bite Biomarkers in Australian Patients
Source: Int J Mol Sci. 2025 Feb 11;26(4):1520. doi: 10.3390/ijms26041520 (PMC11855657; doi:10.3390/ijms26041520)
Supplement: Supplementary file 1 [file ijms-26-01520-s001.zip › Combined.Supplemental Material.20Nov2024.pdf]

Local skin and systemic blood signatures in Australian tick-bitten patients share biological signals.

Table S1. Participant details: Participant de-identified ID, information, tick bite information and relevant case notes. The participants in this study represent a subset of those reported in a paper we have previously published (Lee et al. 2024) and this table was included to ensure critical participant details pertaining to the interpretation of results are readily available.

Participant ID	Sex/ Age	Tick bite information	Case Notes
**AHE**	Male 22	- 5+ past tick bites<br>- Tick attached for 6-24 hours<br>- Tick instar: Nymph	- Blood culture strong positive to ds tick virus (XTC-2) at 1 week post tick-bite.<br>- *93% *Rickettsia* (biting tick)
**AHL**	Male 58	- 4 past tick bites<br>- Tick attached for >24 hours<br>- Tick instar: Adult female	- pre-existing antibodies to Spotted Fever Group *Rickettsia*.<br>- Seroconversion to *Coxiella burnetii* at 3 months post tick-bite<br>- *2% *Rickettsia* (tick bitten skin biopsy)<br>- *94% *Rickettsia* (biting tick)
**AHN**	Female 56	- 5 past tick bites<br>- Tick attached for >24 hours<br>- Tick instar: Nymph	- Redness around the bite<br>- *27% *Rickettsia* (tick bitten skin biopsy)<br>- *59% *Rickettsia* (biting tick)
**AHR**	Male 67	- 4 past tick bites<br>- Tick attached for >24 hours<br>- Tick instar: Adult male	- Redness around the bite<br>- Itchiness<br>- "I appear to develop gut discomfort following a tick bite."<br>- *17% *Rickettsia* (biting tick)
**AJS**	Male 65	- 5+ past tick bites<br>- Tick attached for >24 hours<br>- Tick instar: Larvae	- Redness around the bite<br>- Itchiness<br>- Pathological liver changes associated with tick bite.<br>- *1% *Rickettsia* (tick bitten skin biopsy)<br>- *44% *Rickettsia* (biting tick)
**AHP**	Female 53	- 5+ past tick bites<br>- Tick attached for >24 hours<br>Tick instar: Nymph	- Redness around the bite<br>- Itchiness<br>- *28% *Rickettsia* (biting tick)

*match based on percentage of reads obtained from 16S rRNA gene sequences; [#]double stranded tick virus; "…" participant-reported symptom(s).

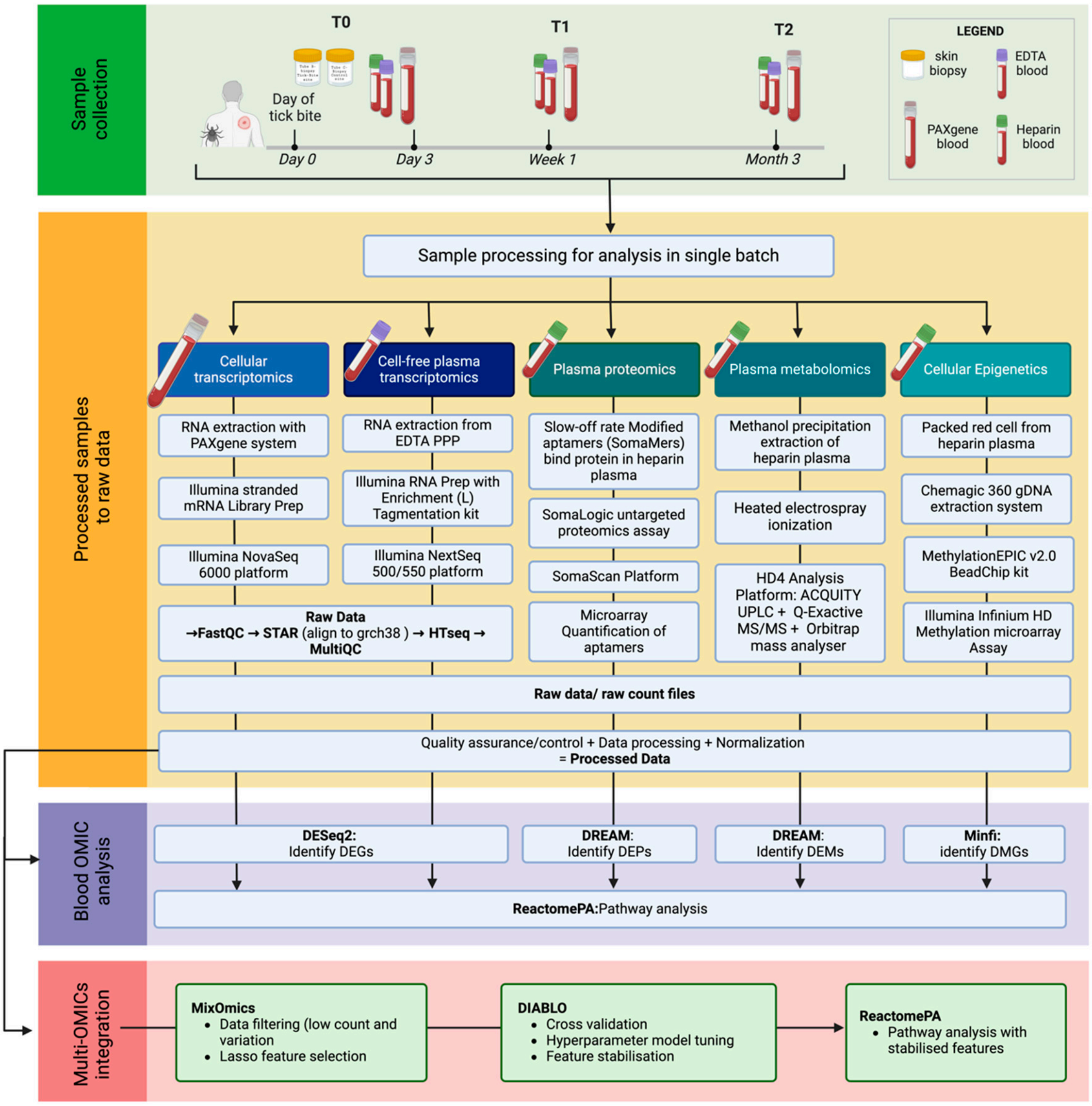

Supplemental Figure S1. Tick-bitten participant sample collection and analysis schedule. Created with BioRender.com

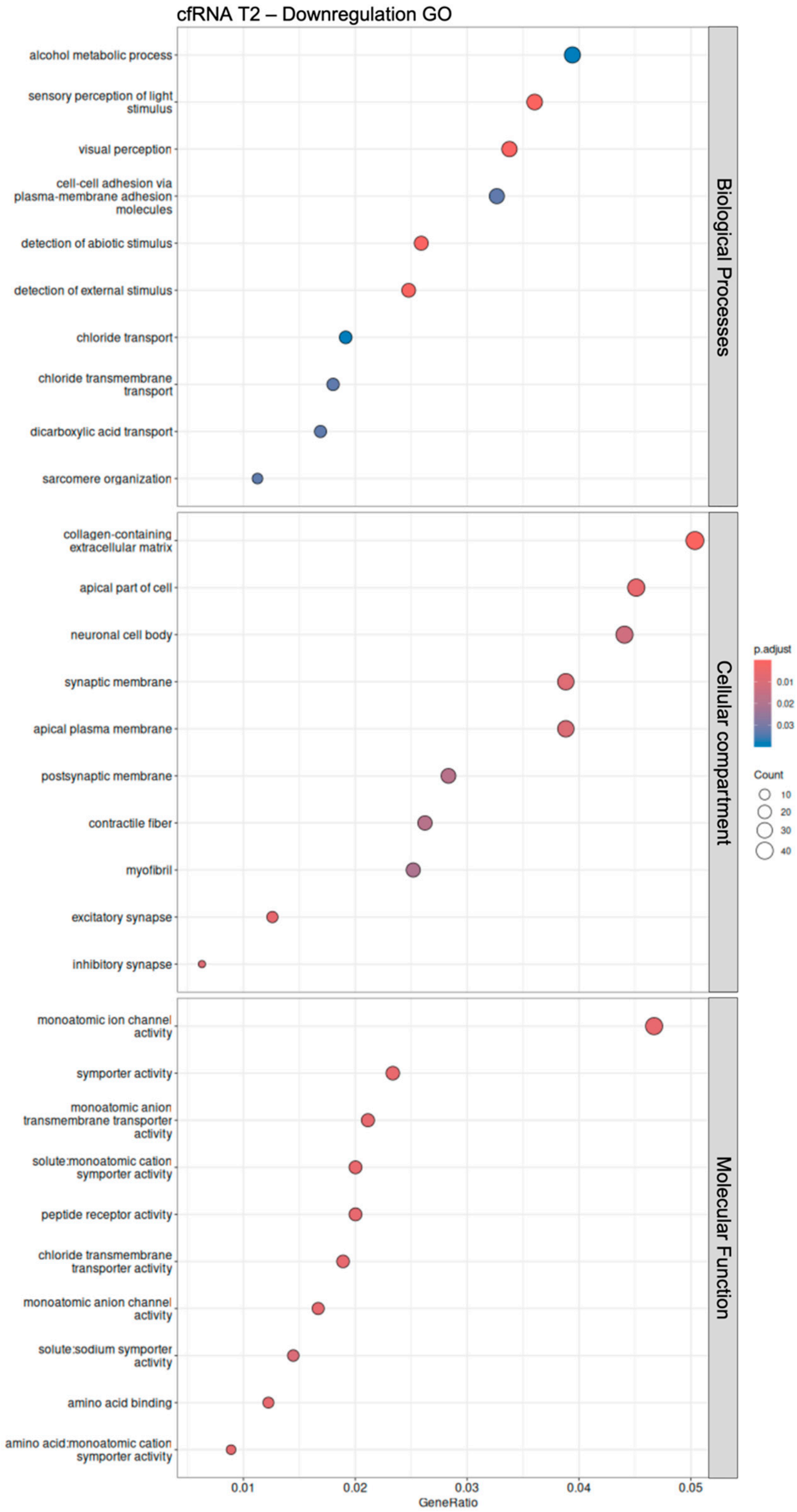

Supplemental Figure S2. Dotplot of downregulated T2 cfRNA in terms of significance in biological processes, cellular compartment and molecular function. Size of the dots represent the number of genes within the gene set while the dot colour represents the enrichment scores based on adjusted p-values.

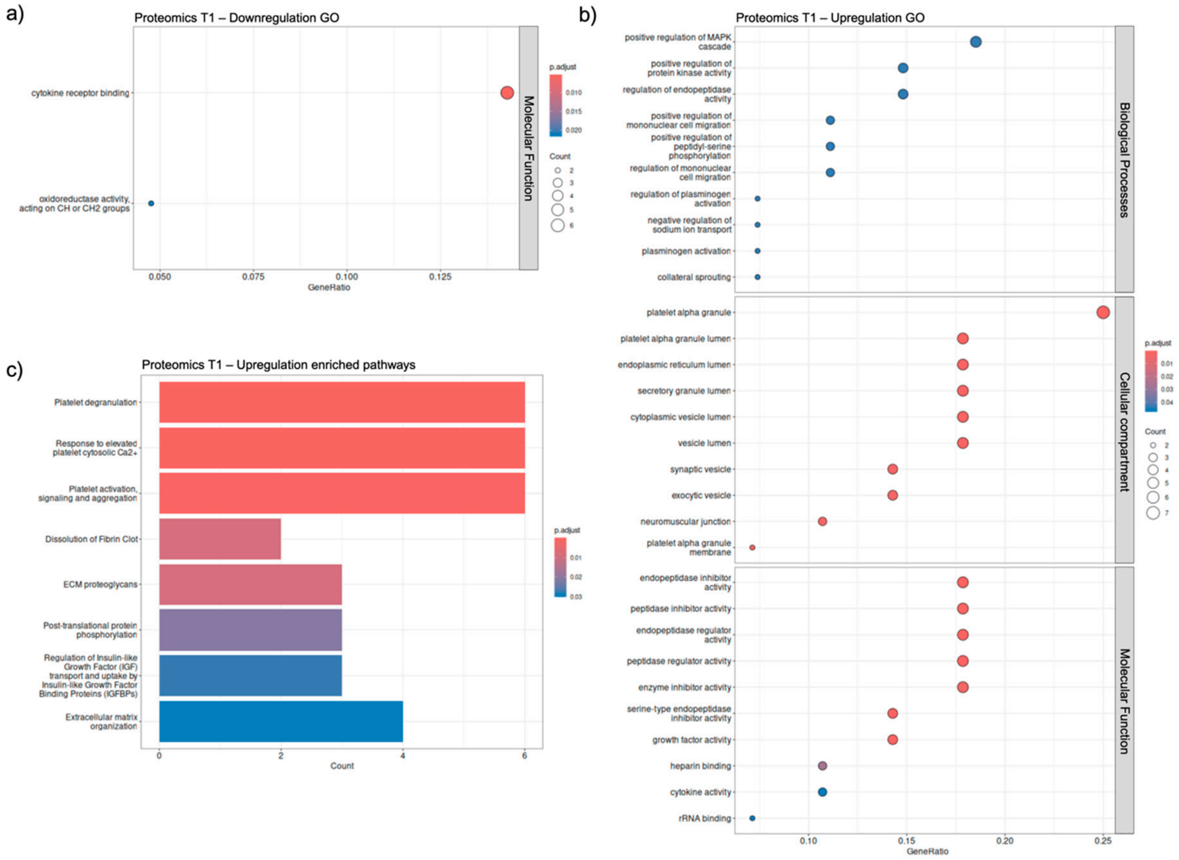

Supplemental Figure S3. Analysis of differentially expressed molecules at T1 as compared to T0. a) Dotplot of downregulated T1 DEPs in terms of significance in biological processes, cellular compartment and molecular function. Size of the dots represent the number of genes within the gene set while the dot colour represents the enrichment scores based on adjusted p-values; b) Dotplot of upregulated T1 DEPs in terms of significance in cellular compartment, molecular function, and biological processes. Size of the dots represent the number of genes within the gene set while the dot colour represents the enrichment scores based on adjusted p-values; c) Enriched pathway analysis of upregulated T1 DEPs in terms of significance based on adjusted p-value represented with the bar colour.

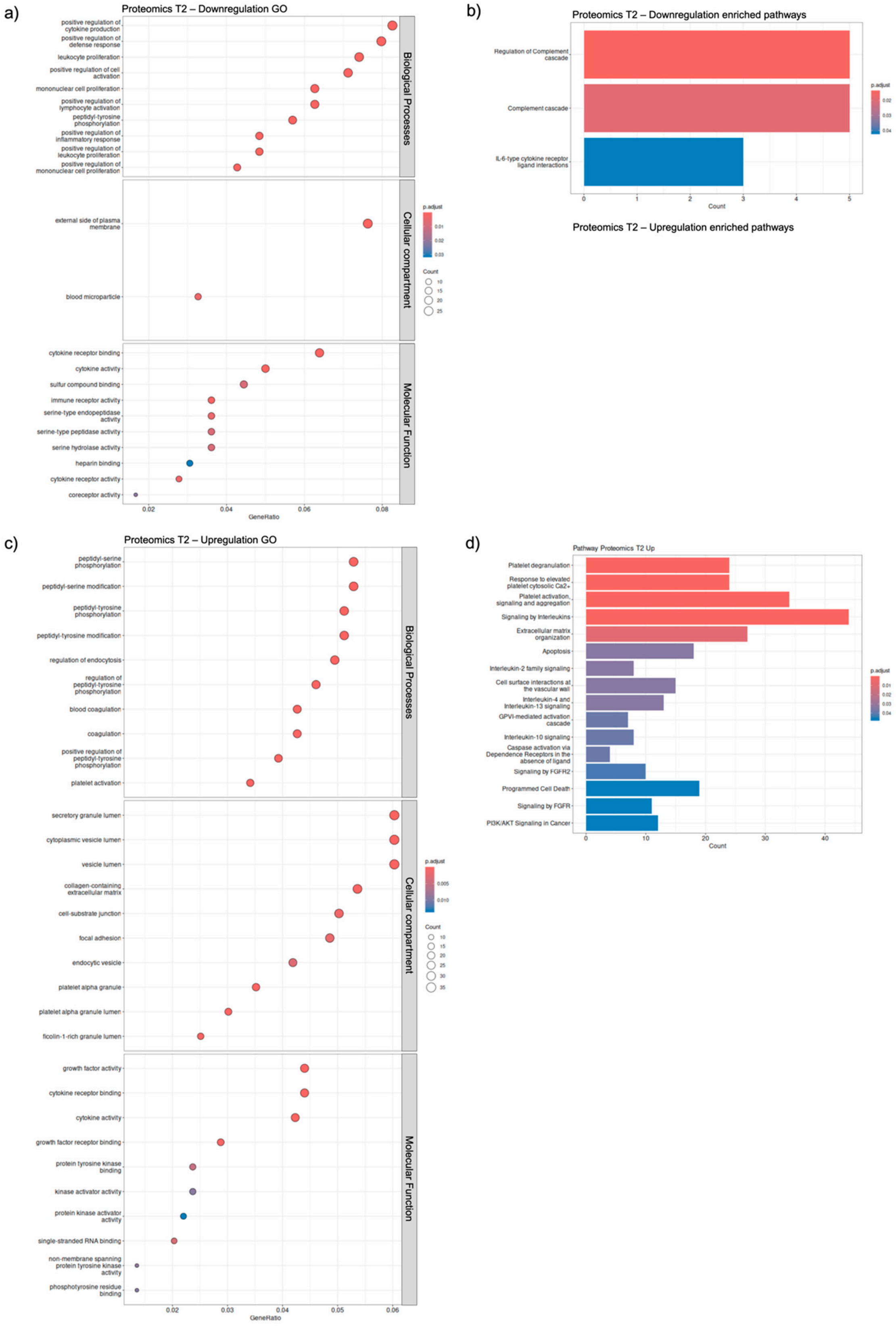

Supplemental Figure S4. Analysis of differentially expressed molecules at T2. a) Dotplot of downregulated T2 DEPs in terms of significance in biological processes, cellular compartment and molecular function. Size of the dots represent the number of genes within the gene set while the dot colour represents the enrichment scores based on adjusted p-values; b) Enriched pathway analysis of downregulated T2 DEPs in terms of significance based on adjusted p-value represented with the bar colour; c) Dotplot of upregulated T2 DEPs in terms of significance in

cellular compartment, molecular function, and biological processes. Size of the dots represent the number of genes within the gene set while the dot colour represents the enrichment scores based on adjusted p-values; d) Enriched pathway analysis of upregulated T2 DEPs in terms of significance based on adjusted p-value represented with the bar colour.

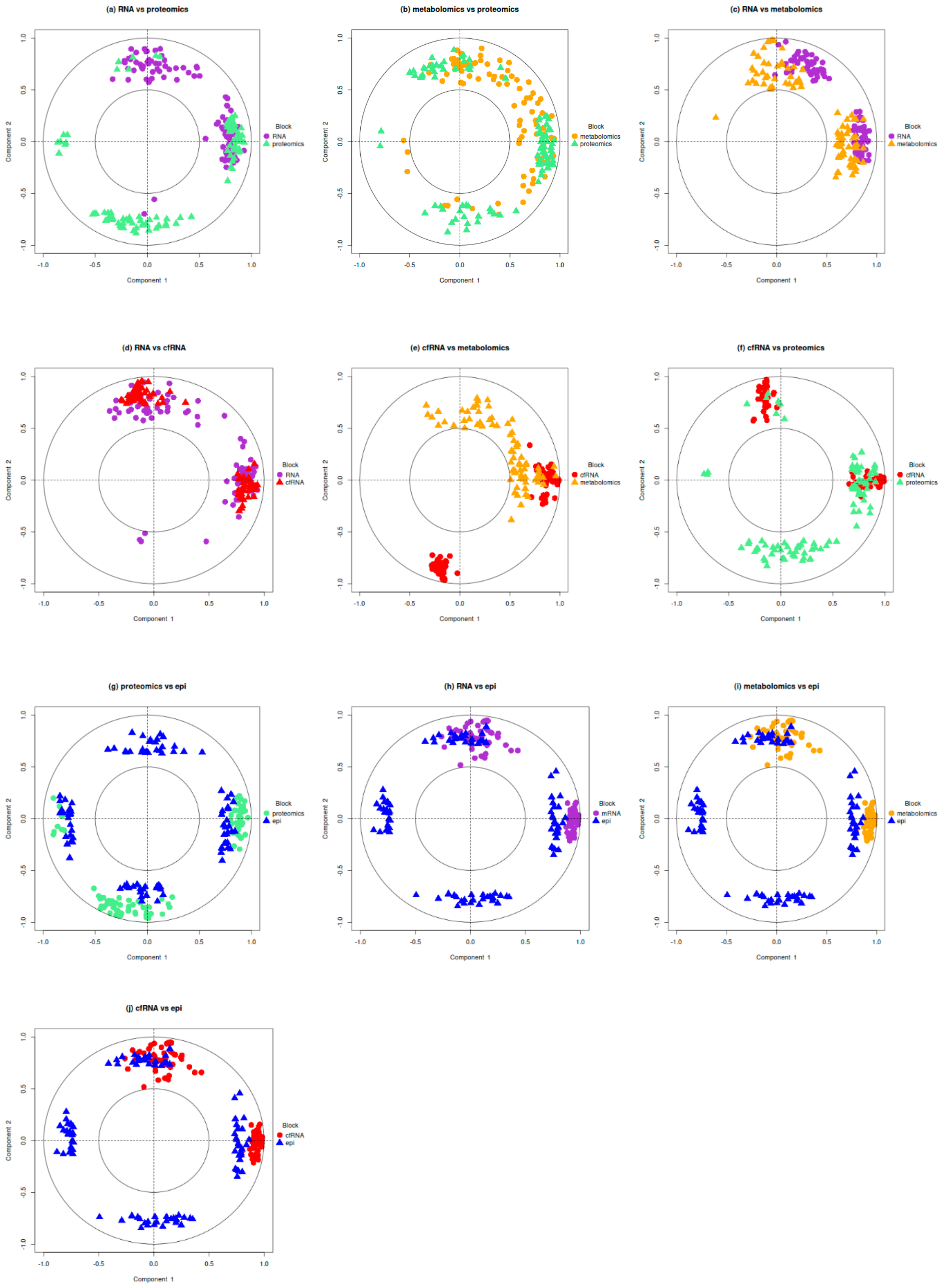

Supplemental Figure S5. Pair-wise correlation circle plot between each OMICs type.

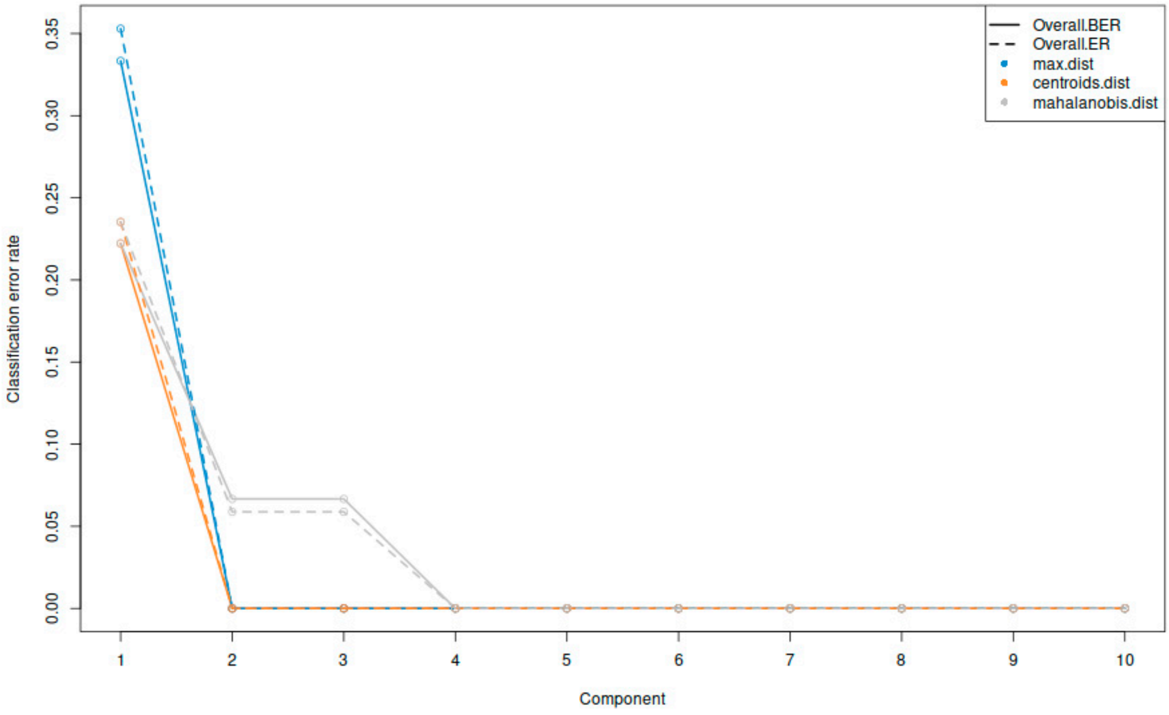

Supplemental Figure S6. DIABLO performance tuning - Classification error rate by component.

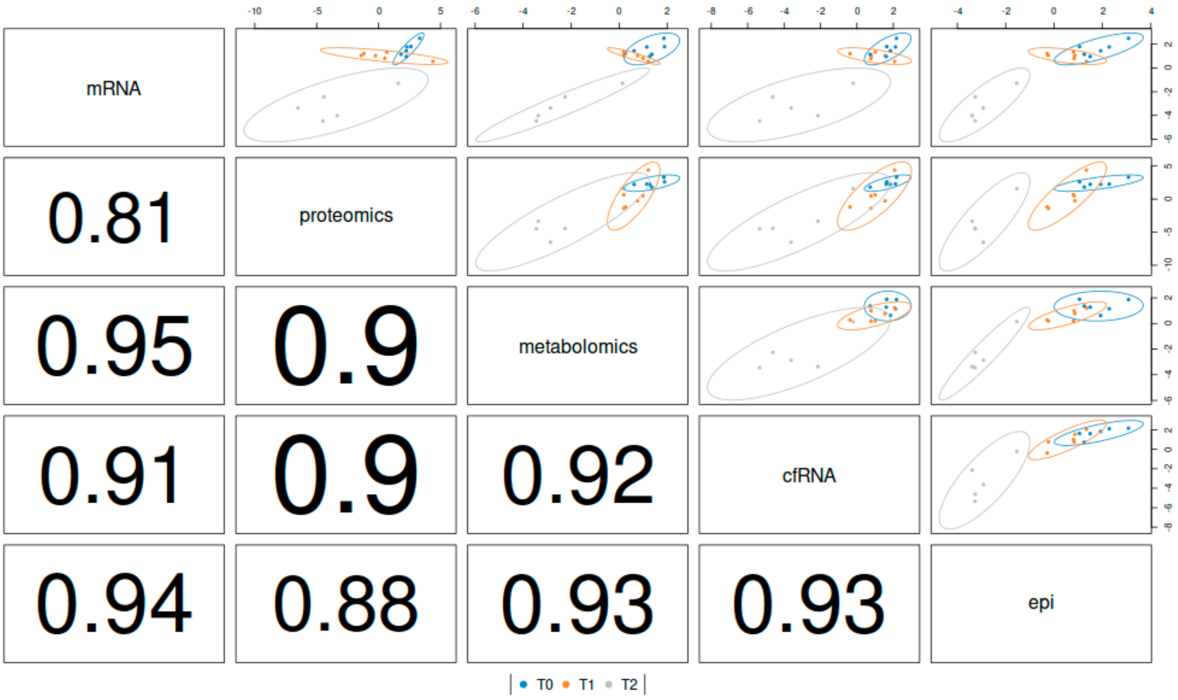

Supplemental Figure S7. DIABLO final model diagnostic plot for component one.

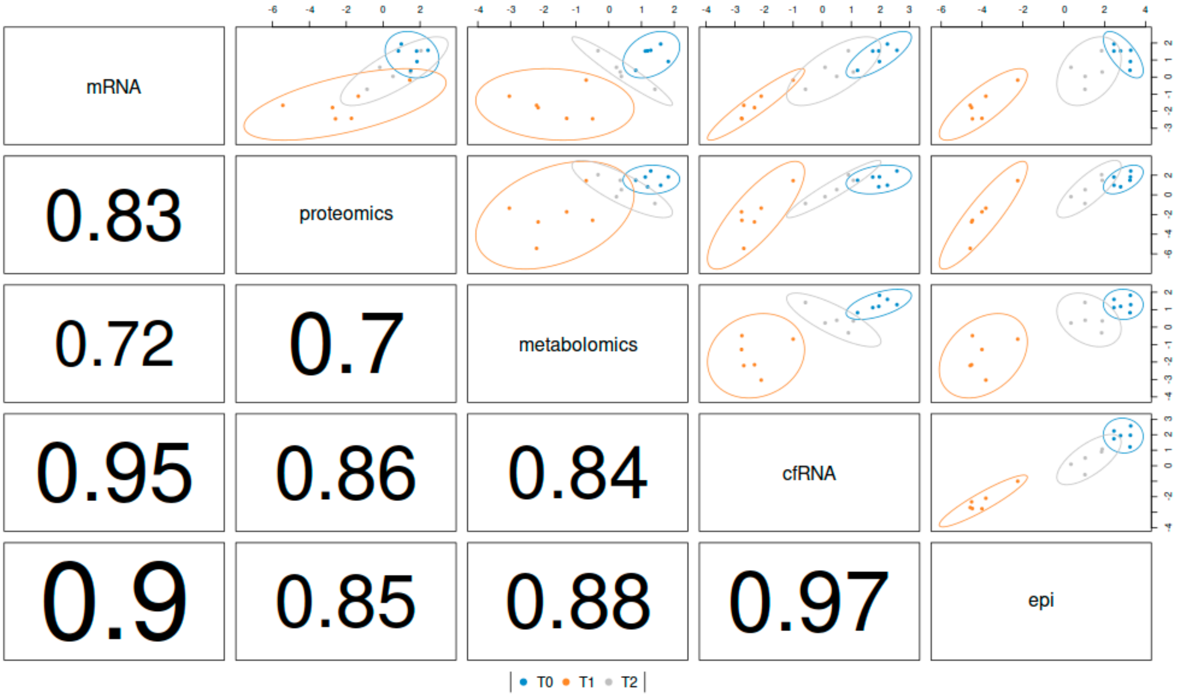

Supplemental Figure S8. DIABLO final model diagnostic plot for component two.

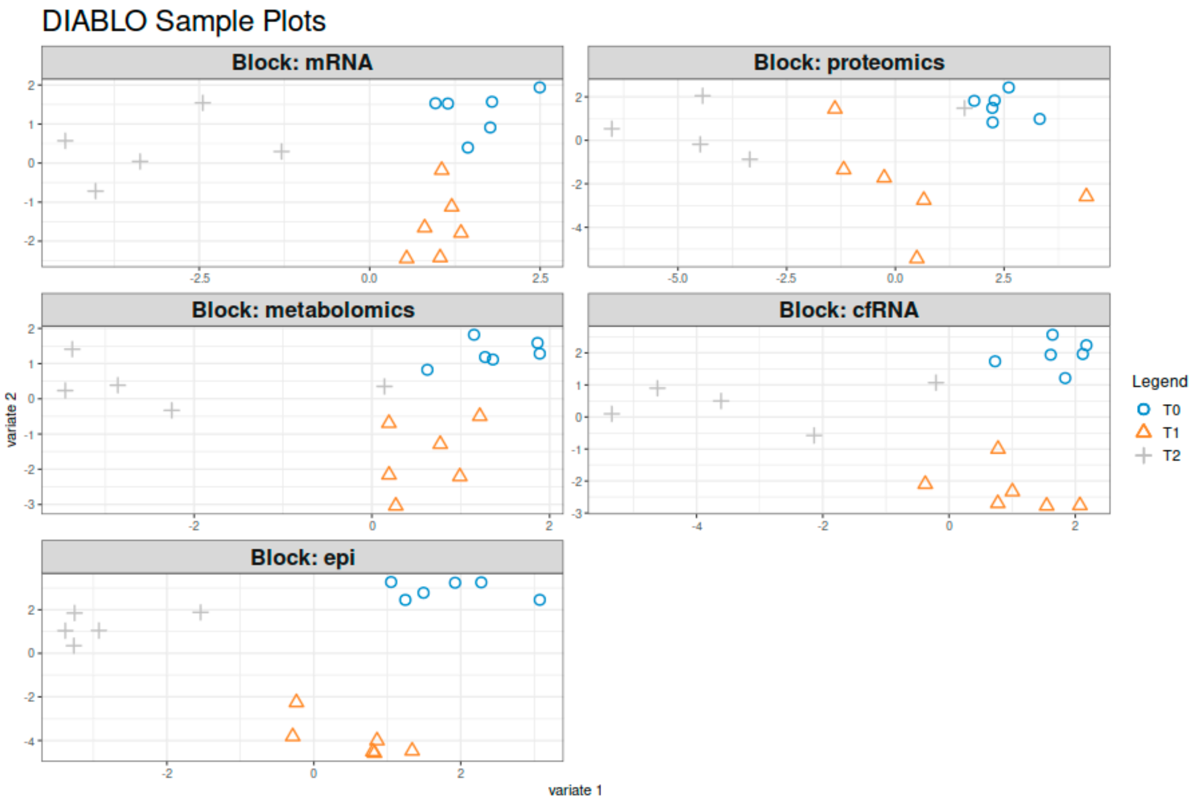

Supplemental Figure S9. DIABLO selected features - Block sample plots.

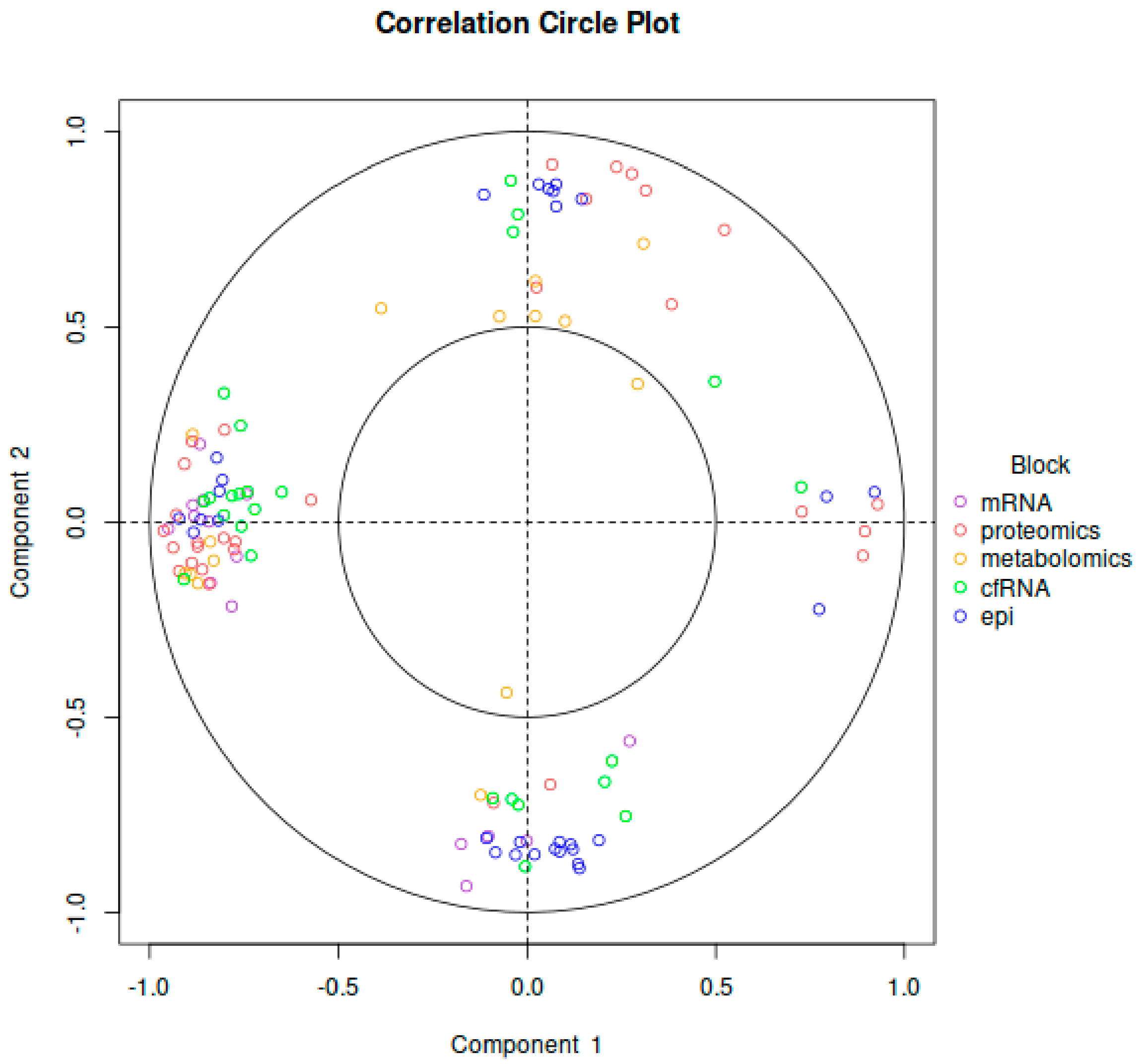

Supplemental Figure S10. DIABLO selected features - correlation circle plot
